# 14-3-3θ phosphorylation at S232 reduces its interactome and regulates axonal trafficking

**DOI:** 10.1242/dmm.052405

**Published:** 2025-12-05

**Authors:** F. Sanders Pair, Rudradip Pattanayak, James A. Mobley, Kyoko Kojima, Mary Gannon, Roschongporn Ekkatine, William J. Stone, Kasandra Scholz, Talene A. Yacoubian

**Affiliations:** ^1^ Center for Neurodegeneration and Experimental Therapeutics, Department of Neurology, University of Alabama at Birmingham, Birmingham, AL 35294, USA; ^2^ Department of Anesthesiology and Perioperative Medicine, Division of Molecular and Translational Biomedicine, University of Alabama at Birmingham, Birmingham, AL 35294, USA; ^3^ Institutional Research Core Program, University of Alabama at Birmingham, Birmingham, AL 35294 , USA

**Keywords:** 14-3-3θ, Affinity-purification mass spectrometry, Axonal trafficking, Parkinson's disease, Dementia with Lewy bodies

## Abstract

14-3-3 proteins impact protein-protein interactions (PPIs) that regulate neuronal functions. The 14-3-3θ isoform is protective in models of Parkinson's disease (PD) and dementia with Lewy bodies (DLB). Human PD and DLB brains show increased 14-3-3θ phosphorylation at S232. To understand the impact of 14-3-3θ phosphorylation on brain PPIs, we performed affinity purification-mass spectrometry using S232 phospho-mutant knock-in mouse models. Proteins binding 14-3-3θ in Cre control cortical lysates were enriched in proteins involved in neuronal morphogenesis and microtubule dynamics. We found a dramatic decrease in proteins binding to 14-3-3θ in S232D mice compared to S232A mice. Axonal trafficking associated with these differentially binding proteins. Live imaging of acidic vesicles in axons revealed reduced net velocity in S232A and S232D neurons compared to that in Cre controls. In S232D neurons, this was due to a dramatic increase in vesicle pausing, while S232A neurons showed reduced segmental velocity, suggesting disrupted dynein motility. We conclude that 14-3-3θ phosphorylation fine tunes axonal transport of acidic vesicles. Disruption of axonal transport with aberrant phosphorylation observed in PD and DLB could contribute to impaired clearance of aggregated proteins in these disorders.

## Teaser

**Summary:** 14-3-3θ phosphorylation disrupts its interactome in mouse brain and causes dysregulation of axonal trafficking, a process that might contribute to impaired clearance of aggregated proteins in neurodegenerative disorders.

## INTRODUCTION

14-3-3 proteins are a family of proteins highly conserved across species consisting of seven mammalian isoforms including β, γ, ε, η, ζ, σ and τ/θ (Zhu et al., 2016; Toker et al., 1992; Ichimura et al., 1988). Through protein-protein interactions (PPIs), 14-3-3s act as a central hub for many different cellular processes, such as cellular trafficking, cytoskeletal dynamics, axonal growth and apoptosis (Pair and Yacoubian, 2021; Antunes et al., 2022; Low et al., 2024). Owing to their extensive PPI networks, 14-3-3 proteins have been associated with a wide range of diseases from cancer to neurodegeneration (Fan et al., 2019; Abdi et al., 2024).

14-3-3s are particularly enriched in the brain, consisting of ∼1% of total brain protein (Martin et al., 1994; Baxter et al., 2002). Our work to date has pointed to a role for the 14-3-3θ isoform in neurodegenerative disorders marked by pathological aggregation of alpha-synuclein (αsyn), such as Parkinson's disease (PD) and dementia with Lewy bodies (DLB). 14-3-3θ interacts with several proteins implicated in PD, including αsyn, LRRK2 and parkin (Kawamoto et al., 2002; Berg et al., 2003; Dzamko et al., 2010; Nichols et al., 2010; Li et al., 2011; Sato et al., 2006), and loss of the ability of 14-3-3θ to regulate and interact with binding partners may promote the neurodegenerative process. Consistent with this, we have observed that 14-3-3θ overexpression is protective in neurotoxin and mutant LRRK2 models, whereas 14-3-3 inhibition accelerates toxicity in these models (Yacoubian et al., 2010; Slone et al., 2011; Ding et al., 2015; Lavalley et al., 2016). Additionally, 14-3-3 inhibition accelerates αsyn internalization, oligomerization and seeding in fibrillar and paracrine αsyn models, whereas 14-3-3θ overexpression protects against these pathological processes (Wang et al., 2018). These findings have also been recapitulated *in vivo*: overexpression of 14-3-3θ delayed αsyn aggregation, ameliorated behavioral deficits and reduced neuronal loss in the preformed fibril (PFF) αsyn model; 14-3-3 inhibition accelerated αsyn aggregation, potentiated behavioral deficits and increased neuronal loss (Underwood et al., 2021).

Given that 14-3-3θ is so abundant in brains, a key unknown is why 14-3-3θ fails to protect against neurodegeneration in PD and related disorders. Our work has shown a dramatic increase in the phosphorylation of 14-3-3θ at serine 232 (S232) in the Triton X-100-insoluble fraction of cortical brain lysates from subjects with PD, DLB and Alzheimer's disease (AD) compared to those from unaffected age- and sex-matched controls (McFerrin et al., 2017). Changes in other phosphorylation 14-3-3 sites were not consistent across multiple neurodegenerative disorders (McFerrin et al., 2017). The increase in S232 phosphorylation was inversely correlated with cognitive function (McFerrin et al., 2017). Further work from our group has shown that the phosphomimetic S232D 14-3-3θ mutant accelerates αsyn aggregation in the αsyn PFF model (Wang et al., 2025). Additionally, the S232D mutant fails to protect in neurotoxin and mutant LRRK2 models (Pattanayak et al., 2024).

The S232 phosphorylation site is limited primarily to the 14-3-3θ isoform, although the T233 phosphorylation site is present in 14-3-3ζ. This phosphorylation site is found in the C-terminal region outside the canonical binding site of 14-3-3θ, but studies suggest that this phosphorylation site can impact PPIs (Dubois et al., 1997). While there is clear evidence that 14-3-3θ interacts with important PD-associated proteins such as αsyn, it has hundreds of binding partners at least in non-neuronal tissue (Segal et al., 2023). Characterization of the 14-3-3θ interactome in the brain has not previously been described. Additionally, how 14-3-3θ phosphorylation at S232 could affect its broader interactome in the brain has not been investigated. Using novel mutant S232 14-3-3θ knock-in (KI) mouse models in combination with affinity purification-mass spectrometry (AP-MS), we investigated the identities of proteins that interact with 14-3-3θ and alterations in these interactions when 14-3-3θ is phosphorylated at S232. Our AP-MS experiment revealed that 14-3-3θ interactors play important roles in neuronal morphogenesis, microtubule dynamics and vesicular transport, along with other roles, while 14-3-3θ phosphorylation reduced binding to proteins important for vesicular trafficking. Live imaging of axonal transport of acidic vesicles in neurons from S232 mutant mouse lines confirmed the importance of 14-3-3θ phosphorylation in the regulation of axonal trafficking.

## RESULTS

### 14-3-3θ interactome in the cortex

**Fig. 1.**
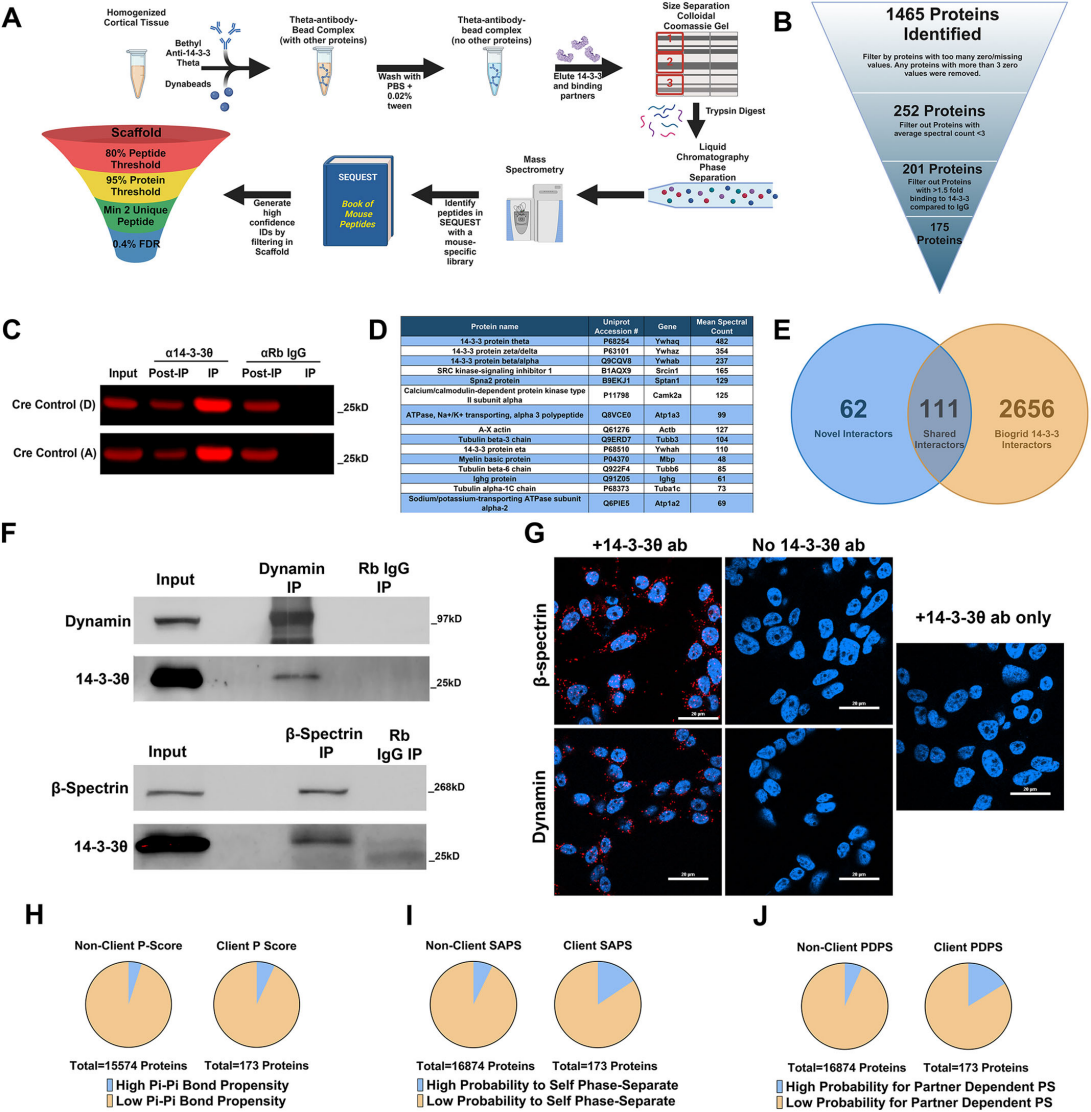
**Identification of novel 14-3-3θ cortical interactors.** (A) Pipeline showing how mouse cortical samples underwent affinity-purification mass spectrometry and peptide identification. FDR, false discovery rate. (B) Protein quality control filtering to identify high-confidence interactors. (C) Representative western blot showing efficient pulldown of 14-3-3θ with minimal 14-3-3θ pulldown in the IgG control pulldowns. (D) Top 15 14-3-3θ interactors based on mean spectral count. (E) Identification of novel 14-3-3θ interactors based on BioGRID's repository of all mammalian 14-3-3 isoform interactors. (F) Western blot of a pulldown for either dynamin or β-spectrin showing successful co-immunoprecipitation of 14-3-3θ. IP, immunoprecipitation. (G) Representative images of proximity ligation assay (PLA) signal between 14-3-3θ:β-spectrin and 14-3-3θ:dynamin in addition to PLA with single antibody controls demonstrating minimal signal. ab, antibody. Scale bars: 20 μm. (H-J) Pie charts showing enrichment of proteins with characteristics that lend themselves to liquid-liquid phase separation, including pScore (H), self-assembling phase separation (SAPS; I) and partner-dependent phase separation (PDPS; J). PS, phase separation. All data come from the PhaSePred database. The database did not have a pi-pi bond propensity for every mouse protein, which is why the total number of proteins in H is reduced compared to that in I and J.

To examine the impact of 14-3-3θ phosphorylation on its protein interactome in the cortex, we used conditional KI S232 mutant mouse lines that we previously created (Wang et al., 2025). As previously described, we generated a conditional phosphomimetic S232D 14-3-3θ mutant KI mouse line and a conditional non-phosphorylatable S232A 14-3-3θ mutant KI mouse line. These mice were crossed with an Emx1-Cre line (Gorski et al., 2002) to induce expression of the S232 mutation in the cortex and hippocampus, two regions impacted in both PD and DLB. To perform AP-MS, we used cortical brain lysates from 3-month-old Emx1-Cre control (Cre control), Emx1-Cre+/− S232A+/+ (S232A) and Emx1+/− S232D+/+ (S232D) mice (Fig. 1A).

We first investigated the general 14-3-3θ protein interactome in the cortex by examination of the proteins that were pulled down with wild-type 14-3-3θ from 12 Cre control mice. After immunoprecipitation with a rabbit polyclonal antibody against 14-3-3θ or rabbit IgG and digestion, trypsin-digested pulldowns underwent liquid chromatography-mass spectrometry. Extra pulldown replicates were run on western blots to assure pulldown quality (Fig. 1C). The resulting peptides were searched using SEQUEST with peptide sequences from an annotated, species-specific library from UniProtKB. Peptide identifications were then filtered in Scaffold for a protein false discovery rate (FDR) of 0.4%.

After filtering proteins for several quality control metrics such as minimum non-zero values, relative amount of binding to IgG controls and minimum spectral count, as described in detail in the Materials and Methods section (Fig. 1B), 175 high-confidence proteins were found to interact with 14-3-3θ in the cortex (Dataset 1). A wide variety of proteins were identified. Unsurprisingly, the top proteins, with regard to spectral count, were other 14-3-3 isoforms; 14-3-3 proteins can form homodimers and heterodimers (Jones et al., 1995). Other top proteins included several cytoskeletal proteins, such as β-spectrin and tubulin subunits (Fig. 1D). Other 14-3-3θ interactors of note included calcium/calmodulin-dependent protein kinase type II subunit alpha (CamKIIα), a sodium-potassium ATPase (Atp1a3) and a heat-shock protein (Hspa8).

We validated several protein interactors identified by AP-MS using co-immunoprecipitation and proximity ligation assay (PLA). 14-3-3θ co-immunoprecipitated with the cytoskeletal protein β-spectrin from wild-type mouse cortical lysates (Fig. 1F). Similarly, 14-3-3θ showed specific co-immunoprecipitation with dynamin 1, a GTPase important for vesicular fission during endocytosis, from wild-type mouse cortical lysates (Fig. 1F). To confirm that these interactions occur within cells and are not an artifact of homogenization of cells allowing proteins to interact in solution, but may never be present naturally in the same subcellular space, we next used PLA techniques to confirm *in situ* protein interactions. SK-N-BE(2)-M17 (M17) neuroblastoma cells demonstrated PLA signal when incubated with antibodies against β-spectrin and 14-3-3θ, but no PLA signal was present when either the anti-14-3-3θ antibody or the anti-β-spectrin antibody was omitted from the reaction (Fig. 1G). Similarly, PLA demonstrated specific interaction between 14-3-3θ and dynamin 1 (Fig. 1G).

We compared the list of 14-3-3θ protein interactors we identified in the cortex to all known binding partners of any mammalian 14-3-3 isoform found in BioGRID's database of protein interactions (Oughtred et al., 2021). At the time of analysis, BioGRID's database contained 2767 unique, previously identified protein binding partners with any of the seven mammalian 14-3-3 proteins. Within our group of 175 proteins, 111 proteins were previously identified in BioGRID's database (Fig. 1E). Sixty-two of the proteins that were found in our list of 14-3-3θ interactors from mouse brain were not identified in BioGRID as known 14-3-3 interactors and were thus identified as novel interactors. Additionally, two of the protein interactors we found were proteins completely absent in the BioGRID database and thus could not be judged as a previously identified 14-3-3 interactor or not.

Previous work has outlined the potential role that 14-3-3 proteins can play in liquid-liquid phase separation (LLPS) (Segal et al., 2023; Liu et al., 2023; Huang et al., 2022), in which membraneless organelles form and regulate cellular functions such as chromatin organization, cytoskeletal rearrangement and autophagic degradation (Zhang et al., 2020). LLPS dysregulation has been implicated to play a role in protein aggregation observed in neurodegenerative disorders (Alberti and Dormann, 2019; Boyko and Surewicz, 2022; Zbinden et al., 2020). We evaluated our 14-3-3θ interactors for protein features that lend themselves to participate in LLPS using the PhaSePred database (Chen et al., 2022). From this database, we pulled out the entire mouse proteome and separated out 14-3-3θ interactors (clients) and the rest of the proteome (non-clients). pScore is the propensity of a protein to participate in pi-pi bonds, which are prevalent in proteins participating in phase separation (Vernon et al., 2018). We found that 6.9% of 14-3-3θ binding partners had a high propensity to form pi-pi bonds compared to 5.0% of non-interacting proteins, using a pScore of 4 as the threshold for high pi-pi bond propensity as defined in Vernon et al. (2018) (Fig. 1H). We also used the PhaSePred database (Chen et al., 2022) with a machine-learning algorithm that predicts a protein's ability to self-assemble to form condensates and participate in LLPS [self-assembling phase separation (SAPS)] as well as their ability to form condensates with partner proteins/RNA [partner-dependent phase separation (PDPS)]. Using a probability score ≥0.7 as the threshold for high probability, 15.6% of 14-3-3θ-interacting proteins had a high probability of participating in SAPS compared to 7.3% of non-interacting proteins (Fig. 1I). Similarly, 16.2% of 14-3-3θ binding partners had a high probability of participating in PDPS compared to 6.7% of non-interacting proteins (Fig. 1J). When we used a more stringent threshold value to set high probability, we saw a similar twofold increase in the percentage of 14-3-3θ binding partners with high probability for self-assembly or partner-dependent assembly.

### 14-3-3θ interactors are involved in neuronal development and morphology

**Fig. 2.**
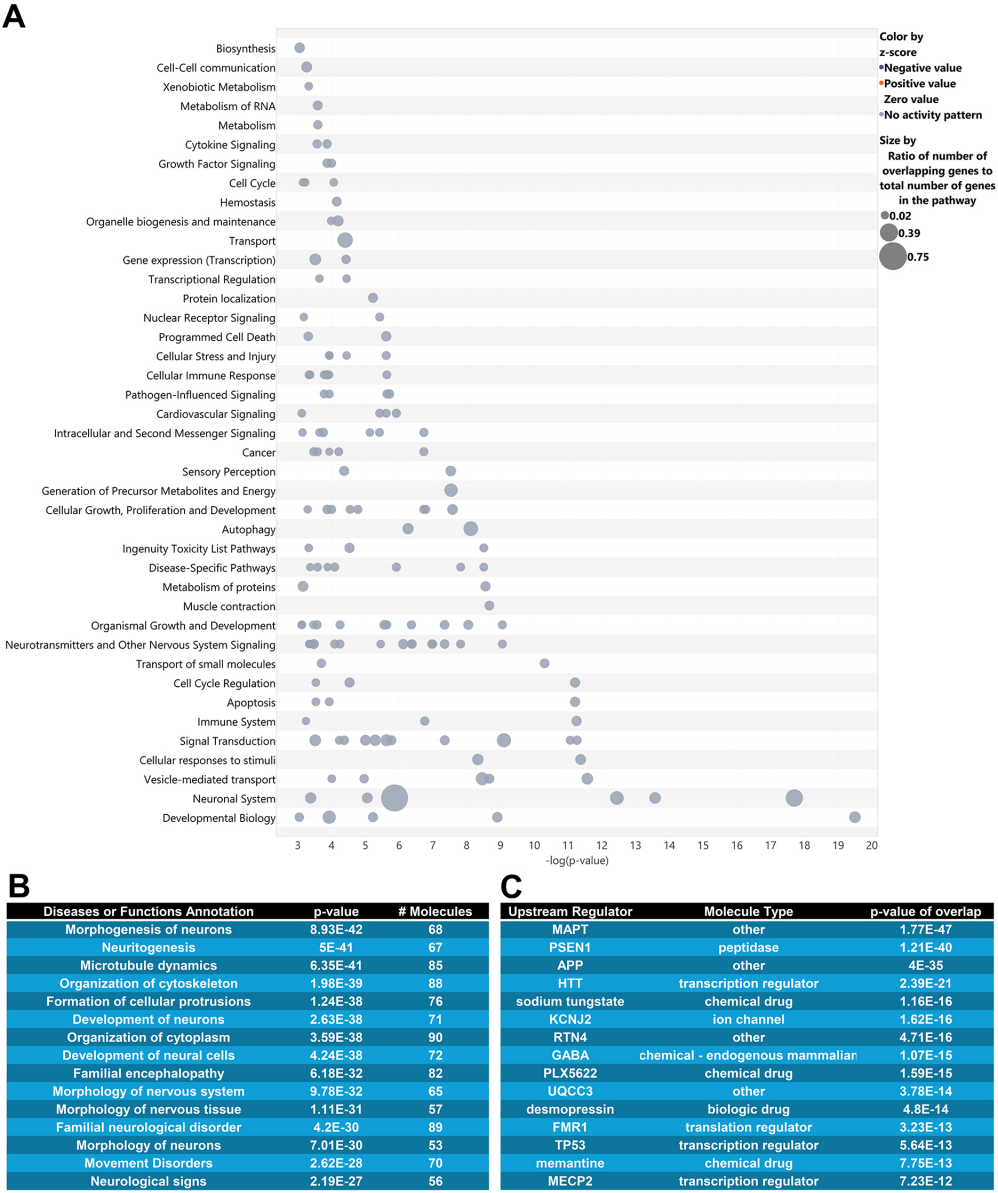
**14-3-3θ interactors are involved in cytoskeletal regulation and neurodegenerative disease.** (A) Ingenuity Pathway Analysis (IPA) canonical pathway analysis of 14-3-3θ interactors, where each circle is a canonical pathway, and each row represents a group descriptor. (B) IPA disease or function annotation shows that 14-3-3θ interactors are mainly involved in cytoskeletal regulation among other roles. (C) IPA upstream regulator analysis shows that key neurodegenerative proteins are highly ranked to regulate 14-3-3θ interactors. All *P*-values were calculated using Fisher's exact test.

We next performed systems analysis on our list of 14-3-3θ interactors using Qiagen's Ingenuity Pathway Analysis (IPA) (Krämer et al., 2014). Developmental biology, neuronal systems and vesicle-mediated transport were among the top pathway groups identified by IPA systems analysis (Fig. 2A). The top five functions identified by IPA's disease and function analysis involved cytoskeletal rearrangement: (1) morphogenesis of neurons, (2) neuritogenesis, (3) microtubule dynamics, (4) organization of cytoskeleton and (5) formation of cellular protrusions (Fig. 2B). Other functions represented by the 14-3-3θ protein interactors included disorder of the basal ganglia and cognitive impairment (Fig. 2B). We also performed upstream regulator analysis in IPA (Fig. 2C). This analysis compares a given protein list to IPA's database of protein regulators, which includes other proteins, toxicants and drugs. IPA calculates the probability of having a similar amount of overlap between a list of proteins within a regulator's set and a list of random proteins that is the same length as the user's list. The top-ranked regulators of our 14-3-3θ-interacting proteins were tau, presenilin-1, amyloid precursor protein (APP) and huntingtin, all proteins involved in neurodegenerative disorders (Fig. 2C).

### Alterations in the interactome of 14-3-3θ secondary to S232 phosphorylation

**Fig. 3.**
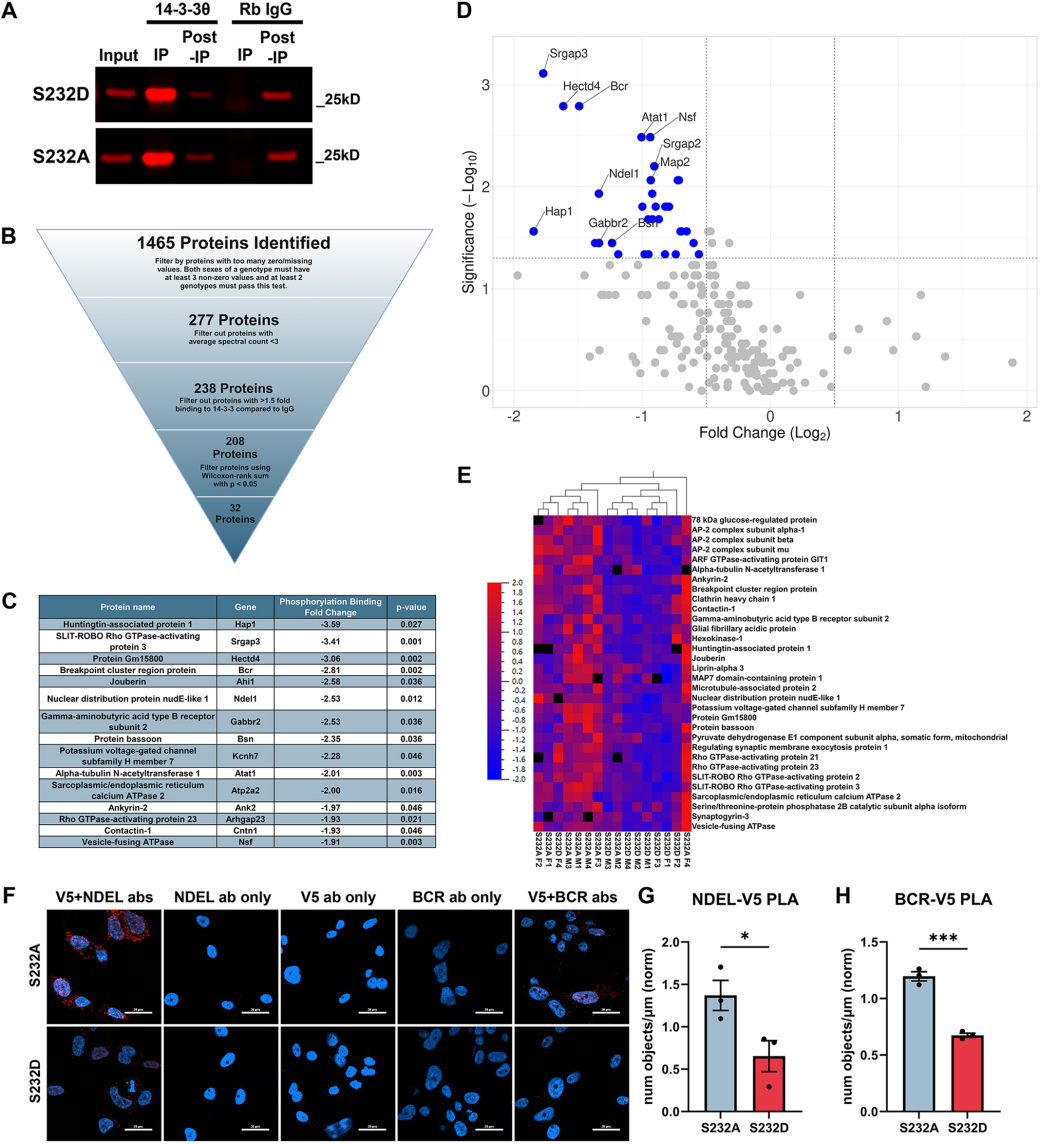
**Phosphorylation-dependent 14-3-3θ interactors demonstrate a profound reduction in binding to the phosphomimetic S232D compared to the non-phosphorylatable S232A.** (A) Representative western blot showing efficient pulldown of 14-3-3θ in both S232D and S232A, with minimal 14-3-3θ pulldown in the IgG control pulldowns. (B) Quality control filtering of identified proteins resulting in 32 proteins with differential binding between S232A and S232D. Wilcoxon rank-sum test, *n*=8 mice per group. (C) The top 15 phosphorylation-dependent interactors ordered by fold change between S232A and S232D. (D) Volcano plot demonstrating the reduction in protein binding to S232D compared to that to S232A. (E) Heatmap of 32 significantly altered proteins. Each protein's abundance is normalized between samples on a scale from −2 to 2. Hierarchical clustering of individual mice show general clustering of S232A and S232D, with female mice showing more variability. (F) Representative images of PLA assays in M17 neuroblastoma cells between V5-tagged phospho-mutants:BCR and V5-tagged mutants:NDEL1. Single antibody controls demonstrate minimal signal. Scale bars: 20 μm. (G) Quantification of V5-14-3-3θ phospho-mutant PLA with NDEL1. *n*=3 independent rounds, unpaired two-tailed *t*-test, *P=0.0482. (H) Quantification of V5-14-3-3θ phospho-mutant PLA with BCR. *n*=3 independent rounds, unpaired two-tailed *t*-test, *P=0.0482, ***P=0.0003. Data are mean±s.e.m.

To determine the potential impact of 14-3-3θ phosphorylation at S232 on 14-3-3θ PPIs, we performed AP-MS on cortical brain samples from S232A (non-phosphorylatable 14-3-3θ mutant) and S232D (phosphomimetic 14-3-3θ mutant) mice. As with protein identification in the Cre control mice, we validated pulldown via western blotting (Fig. 3A) and filtered proteins based on missing values, average spectral count and relative binding to IgG control pulldowns as described in detail in the Materials and Methods section (Fig. 3B); 208 proteins passed these filters between groups (File S2).

To determine whether proteins are preferentially bound to the S232A or S232D 14-3-3θ mutant, we performed a Wilcoxon rank-sum test between S232A and S232D protein interactors, where a protein must have a *P*≤0.05, resulting in 32 proteins that passed our final statistical filter (Fig. 3C; Dataset 2). All 32 proteins significantly different in binding between S232A and S232D showed a higher affinity for S232A than for S232D, as demonstrated by the volcano plot in Fig. 3D. Hierarchical clustering was performed on the S232A and S232D mice to generate a heatmap (Fig. 3E), which again shows the higher propensity for protein binding to the S232A mutant than to the S232D mutant.

To validate our AP-MS findings comparing S232A to S232D mice, we next performed PLA for several proteins that showed differential interaction with S232A versus S232D, including nuclear distribution protein nude-like 1 (NDEL1) and breakpoint cluster region (BCR). NDEL1 is an adaptor protein for the dynein motility complex and has been shown previously to interact with other 14-3-3 isoforms (Chapman et al., 2019; Taya et al., 2007; Mahale et al., 2016; Pandey and Smith, 2011). BCR is classically associated with chronic myelogenous leukemia, in which it forms a fusion protein with tyrosine-protein kinase ABL1 (c-Abl) leading to aberrant kinase activity (Rowley, 1973). To test for interaction specifically between the 14-3-3θ phospho-mutants with NDEL1 or BCR, we used stably transfected M17 cell lines that expressed V5-tagged 14-3-3θ S232A or S232D mutants. This allowed us to use an anti-V5 antibody against the 14-3-3θ phospho-mutants instead of an anti-14-3-3θ antibody, which would detect endogenous wild-type 14-3-3θ along with the phospho-mutants. PLA signal using both anti-V5 and anti-NDEL1 antibodies was about twofold higher in S232A-expressing M17 cells than in S232D-expressing M17 cells (Fig. 3F,G). Minimal PLA signal was detected when either the anti-V5 or anti-NDEL1 antibody was omitted from the reaction (Fig. 3F). PLA signal using both anti-V5 and anti-BCR antibodies was also significantly higher in S232A-expressing cells than in S232D-expressing cells (Fig. 3F,H). No PLA signal was detected when omitting the anti-BCR antibody (Fig. 3F). Minimal PLA signal was present when using NDEL1:V5 antibody pairs and BCR:V5 antibody pairs in naïve M17 cells lacking expression of V5-tagged constructs (Fig. S1). Antibodies against NDEL1 and BCR were validated using shRNA knockdown against either protein: NDEL1 or BCR signal was dramatically reduced in M17 cells in which NDEL1 or BCR was knocked down (Fig. S2).

**Fig. 4.**
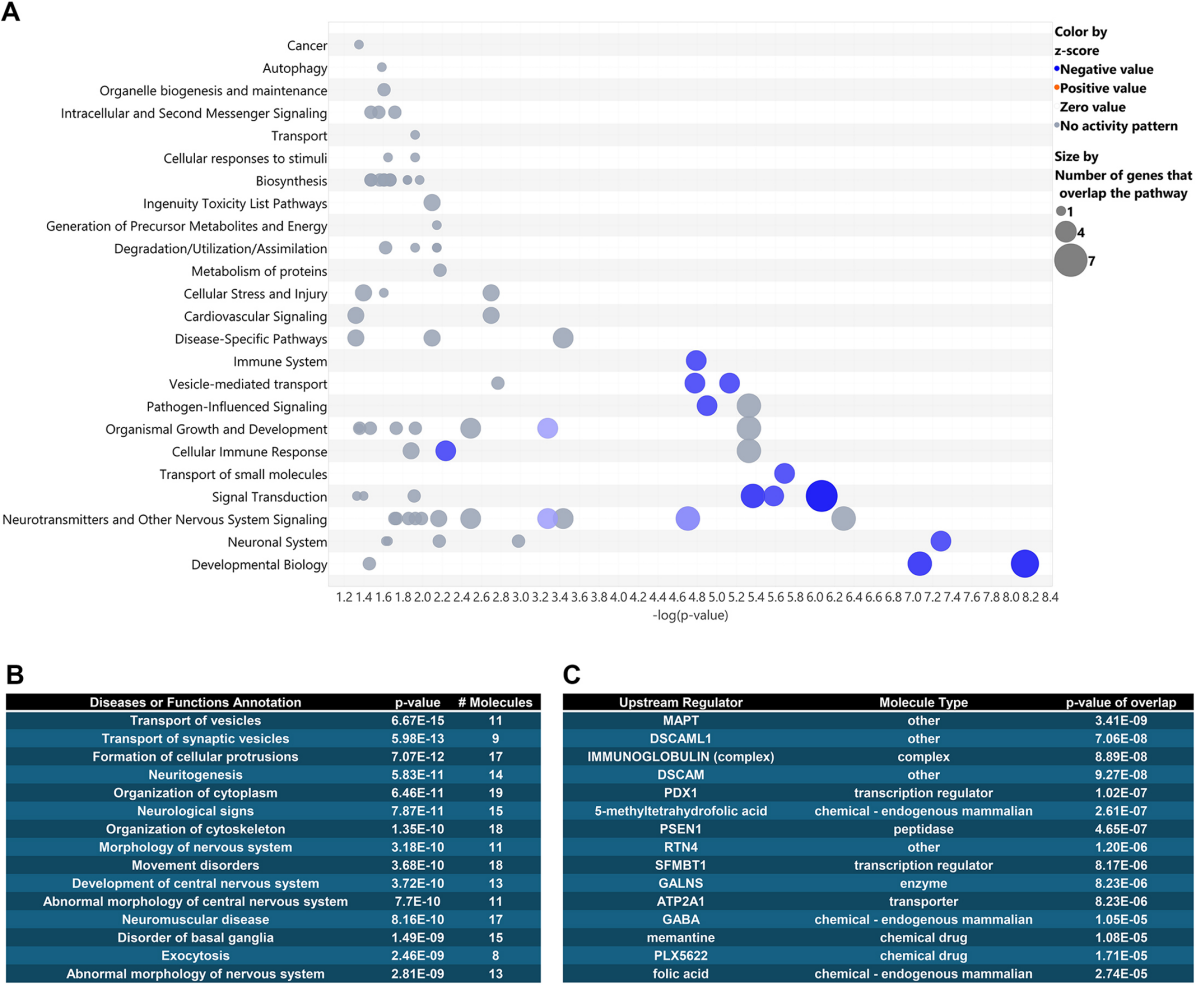
**Phosphorylation-dependent 14-3-3θ interactors are involved in a wide variety of functions, including vesicular transport, and are regulated by neurodegenerative proteins.** (A) IPA canonical pathway analysis of phosphorylation-dependent 14-3-3θ interactors, where each circle is a canonical pathway, and each row represents a group descriptor. Z-score is a relative score of how phosphorylation affects the binding of 14-3-3θ to proteins within that pathway. (B) IPA disease or function annotation shows that phosphorylation at S232 affects the role of 14-3-3θ in vesicular trafficking pathways in addition to cytoskeletal regulation. (C) Upstream regulator analysis of phosphorylation-dependent 14-3-3θ interactors shows that these interactors are regulated by the neurodegenerative proteins tau and presenilin.

To determine what pathways and functions may be impacted by 14-3-3θ phosphorylation, we again performed IPA's canonical pathways and disease and function analyses on the 32 protein interactors that differed between S232A and S232D mice. Once again, top canonical pathways included groupings such as neuronal system, developmental biology and vesicle-mediated transport (Fig. 4A). We found that proteins with preferred interaction with S232A over S232D were enriched in several similar functions as the general 14-3-3θ interactors, such as ‘neuritogenesis’, ‘organization of cytoskeleton’ and ‘morphology of the nervous system’. Interestingly, the highest-scoring two functions were ‘transport of vesicles’ and ‘transport of synaptic vesicles’ (Fig. 4B). Upstream regulator analysis revealed that top-ranked regulators of proteins differentially interacting with S232A versus S232D 14-3-3θ included tau and presenilin, two key proteins associated with neurodegeneration (Fig. 4C). Furthermore, we added our 32 phospho-dependent proteins and αsyn into IPA's pathway network generation tool, and IPA generated an overlay of all experimentally connected protein interactions. Our phospho-dependent proteins showed a dense inter-connectedness with αsyn, the key protein implicated in PD and DLB (Fig. S3).

### S232 phosphorylation regulates axonal trafficking of acidic vesicles

Our functional analysis of the 32 proteins that were affected by S232 mutation pointed to vesicular transport as a key biological function that may be affected by phosphorylation at this site. Several 14-3-3 proteins have been shown to interact with motor transport proteins and their adaptors required for both anterograde and retrograde transport within axons (Geiger et al., 2014; Chapman et al., 2019; Mendoza et al., 2024; Li et al., 2023; Taya et al., 2007; Pandey and Smith, 2011). We found that several proteins showing decreased interaction with S232D in our AP-MS study included key proteins involved in axonal trafficking, such as the dynein complex adaptor NDEL1, bassoon and huntingtin-associated protein 1 (HAP1), among others.

**Fig. 5.**
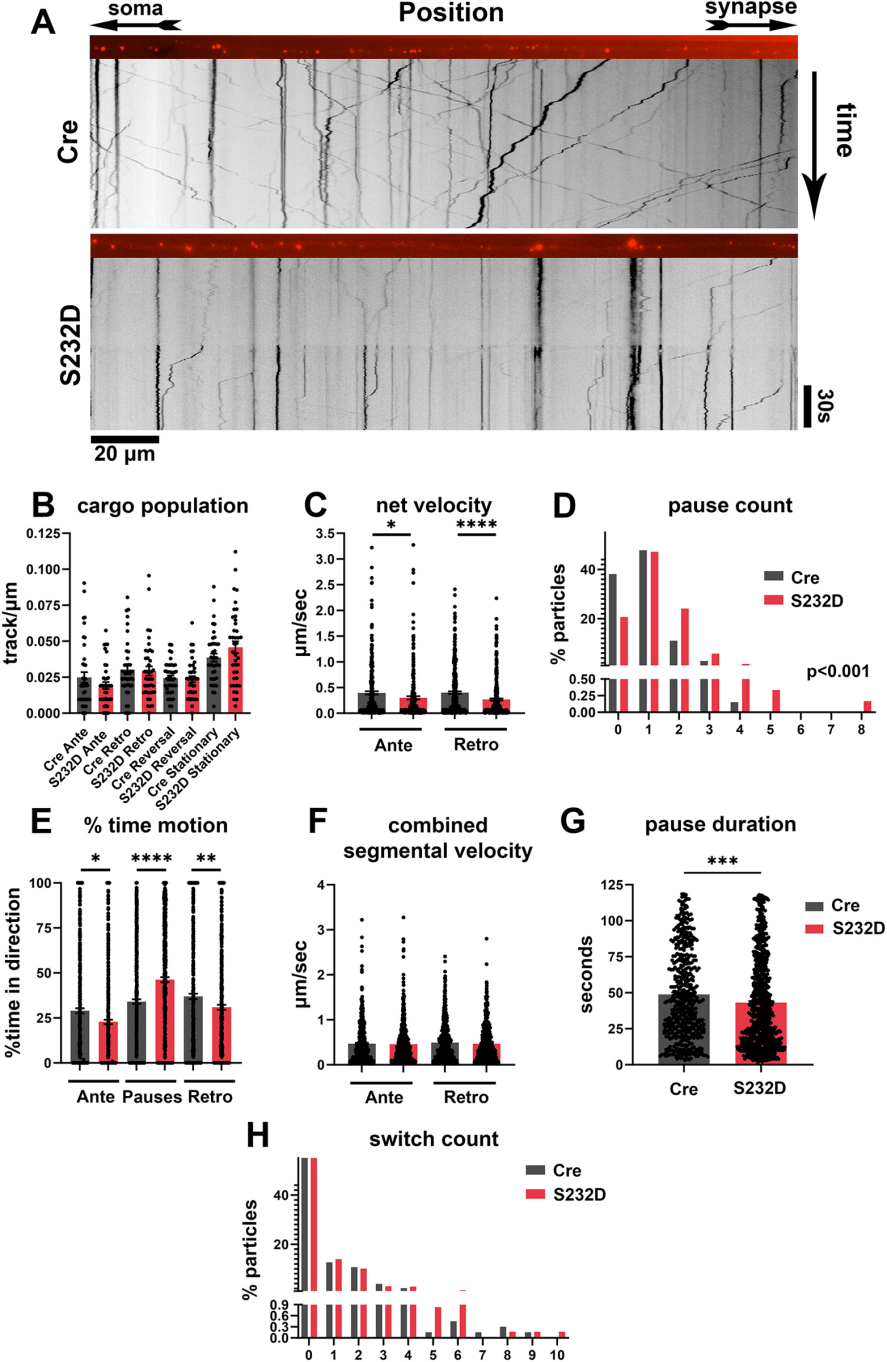
**Acidic vesicles in S232D mouse neurons show axonal trafficking deficits.** (A) Representative kymographs and axons from S232D and Cre control mice. (B) Number of acidic vesicles moving in the anterograde, retrograde, reversal and stationary directions in S232D and Cre control axons, *n*=3 independent rounds with 40 axons per group, Mann–Whitney test (anterograde, retrograde) or unpaired two-tailed *t*-test (reversal, stationary). (C) Net velocity in anterograde and retrograde directions in S232D and Cre control axons, *n*=3 independent rounds with 527 and 702 total vesicles, respectively, Mann–Whitney test. (D) Pause counts in S232D and Cre control axons, *n*=3 independent rounds with 1261 total vesicles, zero-inflated count model, *P*<0.001. (E) Percent time motion in the anterograde, retrograde and paused motion in S232D and Cre control axons, *n*=3 independent rounds with 1261 total vesicles, Mann–Whitney test (anterograde, paused) or unpaired two-tailed *t*-test (retrograde). (F) Anterograde or retrograde combined segmental velocity in S232D and Cre control axons, *n*=3 independent rounds with *n*=1027 and 1259 total segments, respectively, Mann–Whitney test. (G) Pause duration in S232D and Cre control axons, *n*=3 independent rounds, with 1261 total vesicles, Mann–Whitney test. (H) Number of direction switches in S232D and Cre control axons. *n*=3 independent rounds with 1261 total vesicles, zero-inflated count model, *P*=0.262. *P<0.05, **P<0.01, ***P<0.001, ****P<0.0001. Data are mean±s.e.m.

To assess how S232 phosphorylation may affect axonal trafficking, we plated primary hippocampal neurons from Cre control and S232D mice in dual-chamber microfluidic devices and then incubated these neurons with 200 μM LysoTracker Red DND-99 dye for 1 h to label acidic vesicles at day *in vitro* (DIV) 10 prior to live imaging of axons (Movies 1, 2). Kymographs were generated and analyzed using the freely available Fiji plugin KymoAnalyzer (Neumann et al., 2017) (Fig. 5A).

We did not observe any significant differences in particles moving in an anterograde, retrograde or reversal direction between Cre control and S232D neurons (Fig. 5B). Although cargo population did not differ, S232D neurons showed a significant reduction in net velocity in both directions: a 23% reduction in anterograde net velocity and a 33% reduction in retrograde net velocity (Fig. 5C). Additionally, S232D neurons had a 54% increase in the number of pauses that occurred in these vesicles in all directions (Fig. 5D). This was also corroborated by a measurement used by KymoAnalyzer called percent time motion, which measures what percentage of the time each acidic vesicle is moving in a particular direction or paused. Compared to Cre controls, S232D neurons had a 21% reduction in anterograde movement, 16% reduction in retrograde movement and 36% increase in stationary time (Fig. 5E).

Notably, there were no significant alterations in combined segmental velocity between Cre control and S232D neurons (Fig. 5F). Combined segmental velocity represents the velocity an acidic vesicle moves at before either pausing or changing direction. Interestingly, S232D neurons showed a 20% reduction in pause duration compared to Cre control neurons (Fig. 5G). Also notable was that there was no significant change in how often acidic vesicles changed direction (switch count; Fig. 5H). Combined, these data suggest that acidic vesicles in the axons of S232D mice pause more frequently, leading to a significant reduction in net velocity.

**Fig. 6.**
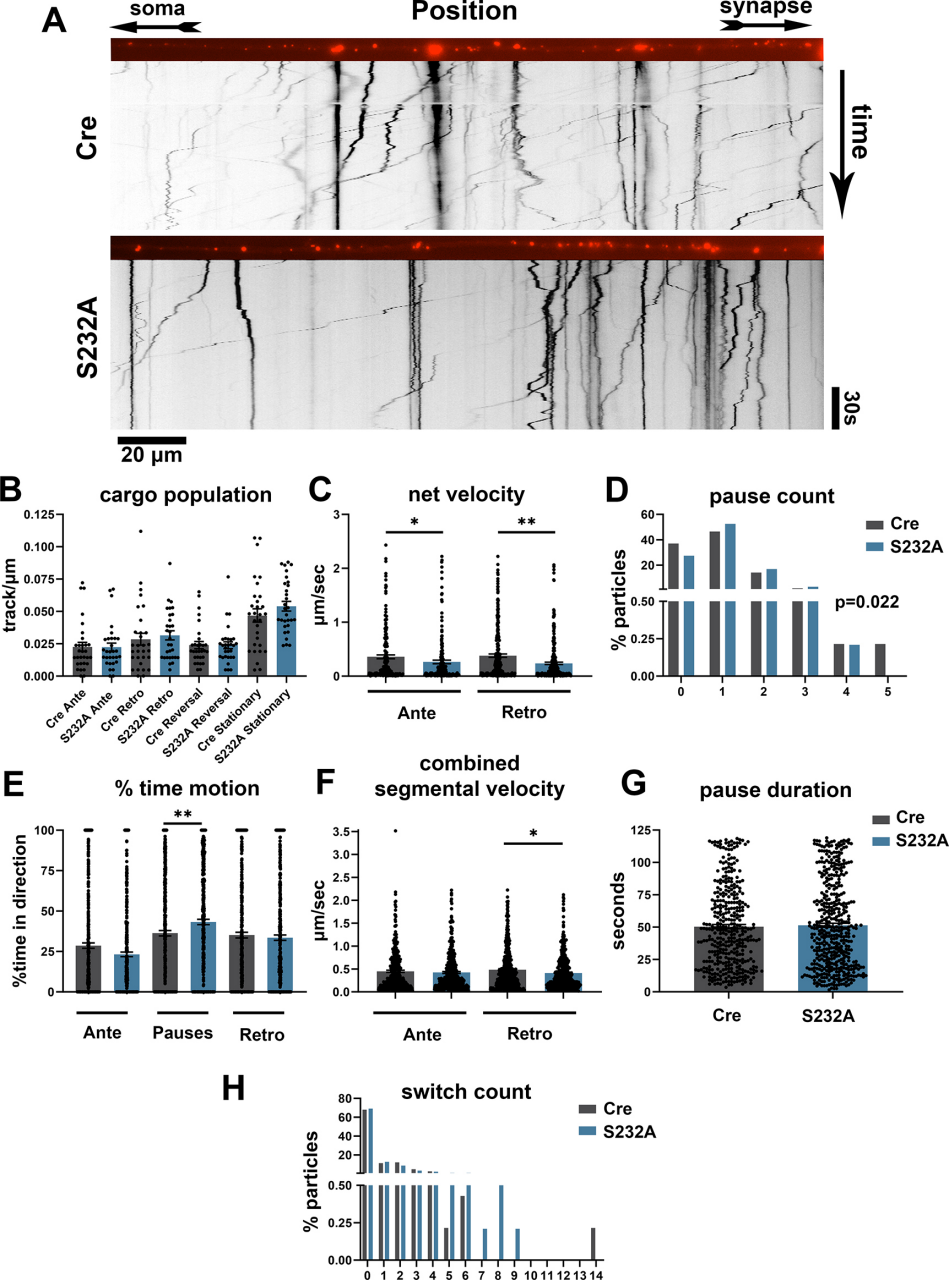
**Acidic vesicles in S232A mouse neurons show axonal trafficking deficits.** (A) Representative kymographs and axons from S232A and Cre control mice. (B) Number of acidic vesicles moving in the anterograde, retrograde, reversal and stationary directions in S232D and Cre control axons, *n*=3 independent rounds with 30 axons per group, Mann–Whitney test (anterograde, retrograde, reversal) or unpaired two-tailed *t*-test (stationary). (C) Net velocity in the anterograde and retrograde direction in S232A and Cre control axons, *n*=3 independent rounds with 388 and 534 total vesicles, respectively, Mann–Whitney test. (D) Pause count in S232A and Cre control axons, *n*=3 independent rounds with 944 total vesicles, zero-inflated count model, *P*=0.022. (E) Percent time motion in the anterograde, retrograde and paused motion in S232A and Cre control axons, *n*=3 independent rounds with 944 total vesicles, Mann–Whitney test. (F) Combined segmental velocity in S232A and Cre control axons, *n*=3 independent rounds with 778 and 883 total segments, respectively, Mann–Whitney test. (G) Pause duration in S232A and Cre control axons, *n*=3 independent rounds with 1261 total vesicles, Mann–Whitney test. (H) Number of direction switches in S232A and Cre control axons, *n*=3 independent rounds with 1261 vesicles, zero-inflated count model, *P*=0.262. *P<0.05, **P<0.01. Data are mean±s.e.m.

Similarly, we examined axonal trafficking using LysoTracker in primary hippocampal cultures from Cre control and S232A neurons (Fig. 6A). S232A neurons showed no significant alteration in the density of acidic vesicles moving in any direction or remaining stationary (Fig. 6B). Acidic vesicles showed a 27% reduction in anterograde net velocity and a 37% reduction in retrograde net velocity in S232A neurons compared to Cre controls (Fig. 6C). Vesicles in S232A neurons showed a small 17% increase in pause count (Fig. 6D). Acidic vesicles showed no significant change in percent time motion in either the anterograde or retrograde direction, but they did show a small 19% increase in the percent time paused in S232A neurons (Fig. 6E). Interestingly, S232A neurons showed a 15% reduction in combined segmental velocity in the retrograde direction (Fig. 6F). This retrograde-specific reduction suggests that there may be some hindrance to the dynein motor complex's ability to move. Acidic vesicles had no significant alteration in pause duration (Fig. 6G) or switch count (Fig. 6H) in S232A neurons compared to Cre control neurons. In summary, compared to those in Cre controls, acidic vesicles in S232A neurons had a significant reduction in net velocity due to a combination of increased pause frequency as well as potential deficits in dynein motility.

## DISCUSSION

Here, we used AP-MS on cortical lysates to characterize the global 14-3-3θ interactome in the brain and additionally to examine the impact of 14-3-3θ phosphorylation at S232 on 14-3-3θ PPIs in the brain. 14-3-3θ interactors in the brain focused on functions related to neuronal development and morphology. Performing AP-MS in S232A and S232D KI mouse cortex, we found a profound reduction in 14-3-3θ binding interactions in S232D mice compared to S232A mice. Any protein that had a significant difference in interaction between our phospho-mutant mice had significantly more interaction with our S232A mice, including key proteins involved in axonal trafficking. Examination of axonal trafficking of acidic vesicles demonstrated that S232 mutations had significant effects on vesicular trafficking, pointing to an important role for 14-3-3θ phosphorylation in the fine tuning of vesicular transport in axons.

To our knowledge, this is the first characterization of the interactome of the 14-3-3θ isoform in the brain. 14-3-3 proteins are known to have hundreds of binding partners and, consequently, be involved in numerous cellular functions (Pair and Yacoubian, 2021; Antunes et al., 2022; Low et al., 2024). Although many functions were associated with our 14-3-3θ interactors, the top functions of these protein interactors centered primarily on cytoskeletal re-arrangement, neuronal morphogenesis and molecular transport. Of note, we are not the first to characterize a 14-3-3 isoform's interactome in the brain. Heverin et al. (2012) performed AP-MS on recombinant his-tagged 14-3-3ζ pulldowns from hippocampal lysates from C57BL/6 adult male mice. Of the 29 proteins identified as 14-3-3 recombinant his-tagged 14-3-3ζ interactors, 17 matches were found in our 14-3-3θ interactome list. Additionally, key biological processes enriched in their protein list overlapped with those functions represented in our 14-3-3θ protein list, including cytoskeletal organization, neuronal migration, axon guidance, microtubule-based movement and synaptic transmission.

Of the 175 high-confidence interactors, 62 of these proteins have not been previously identified to interact with any 14-3-3 mammalian isoform in BioGRID's database (Oughtred et al., 2021). Additionally, using computational approaches, we found that 14-3-3 protein interactors are enriched in characteristics that lend themselves to participate in LLPS, which is consistent with the work of Segal et al. (2023), showing that 14-3-3s regulate aggregation and phase separation of binding partners. Using several characteristic metrics, our high confidence 14-3-3θ interactors had a higher ratio of proteins with LLPS characteristics compared to the mouse proteome. Given the increasing evidence for the role of LLPS in neurodegenerative disorders (Alberti and Dormann, 2019; Boyko and Surewicz, 2022; Zbinden et al., 2020), our findings suggest a potential role for 14-3-3θ in the regulation of LLPS to prevent neurodegeneration. Liu et al. (2023) previously showed 14-3-3ζ to regulate LLPS of phosphorylated and glycated tau and thus to affect its physiological and pathological functions. Interestingly, removal of the C-terminal tail of 14-3-3ζ caused it to lose its ability to regulate the LLPS of tau. The analogous phosphorylation site on 14-3-3ζ to S232 on 14-3-3θ is T233, which is in the C-terminal tail. This points to the potential role S232 phosphorylation may play in regulating 14-3-3θ-mediated LLPS. Future studies are needed to investigate the impact of 14-3-3θ and its phosphorylation on LLPS of proteins implicated in neurodegeneration, such as tau.

Previously, we have shown that, compared to samples from unaffected age-matched controls, Triton X-100-insoluble cortical samples from patients with PD and DLB have increased 14-3-3θ phosphorylation at S232 (McFerrin et al., 2017). Because 14-3-3θ has been shown to interact with several proteins implicated in PD, including αsyn and LRRK2 (Berg et al., 2003; Dzamko et al., 2010; Kawamoto et al., 2002; Li et al., 2011; Nichols et al., 2010), phosphorylation of 14-3-3θ may impact toxicities related to these proteins. Given our previous work showing the ability of 14-3-3θ to reduce αsyn aggregation and toxicity, we recently demonstrated that the S232D phosphomimetic showed reduced binding to αsyn and accelerated αsyn aggregation (Wang et al., 2025). Similarly, the S232D phosphomimetic failed to reduce LRRK2 kinase activity and toxicity, as both wild-type and S232A 14-3-3θ can (Pattanayak et al., 2024). However, because 14-3-3θ has such a wide array of protein interactors, it is critical to understand other PPIs that may be disrupted by 14-3-3θ phosphorylation that could contribute to the pathological process in neurodegenerative disorders marked by increased 14-3-3θ phosphorylation. Using a combination of AP-MS with our phospho-mutant mouse models to investigate what other interactions may be affected, we found a striking difference in protein interactions with the S232A and S232D mutants. Thirty-two proteins had a significant difference in interaction with these S232 mutants. Every single protein interacted more with S232A than with S232D. Key functions associated with those proteins that preferentially bound S232A included cytoskeletal rearrangement and microtubule dynamics, and the highest scoring function was vesicular transport, which is known to be disrupted in neurodegenerative disorders (Cheng et al., 2011; Vilarino-Guell et al., 2011; Egan et al., 2012; Wang et al., 2014; Sleigh et al., 2019; Farrer et al., 2009; Stokin et al., 2005; Chu et al., 2012; Ferguson, 2018). Additionally, IPA's upstream regulator analysis revealed that several regulators of the proteins with differential interaction between S232A and S232D include proteins implicated in neurodegenerative diseases, including tau and presenilin. These data reinforce the notion that S232 phosphorylation contributes to disease pathology.

We identified several trafficking proteins, such as NDEL1, bassoon and HAP1, which had significantly more interaction with S232A than with S232D. This differential interaction suggested that 14-3-3θ phosphorylation could regulate axonal trafficking. To test this, we examined axonal trafficking of acidic vesicles in primary neurons from Cre control, S232A and S232D mice. Compared to their respective Cre controls, both S232A and S232D neurons demonstrated axonal trafficking changes, yet the extent and magnitude of trafficking defects were more prominent in S232D neurons. Net velocity of acidic vesicles in both anterograde and retrograde directions were significantly reduced in both S232D and S232A neurons. Increased pausing of acidic vesicles was observed in both S232D and S232A neurons compared to that in their Cre controls, yet pausing was much more dramatically increased in S232D neurons (57% reduction in S232D versus 17% in S232A neurons). Despite similar changes in net velocity in S232A and S232D neurons, several other notable differences suggest that different mechanisms underlie the impact of S232A and S232D mutations on acidic vesicle transport. In the case of S232D neurons compared to Cre controls, we observed a decrease in percent time in motion in anterograde and retrograde directions with a 36% increase in percent time stationary, while no impact was seen in percent time in anterograde and retrograde motion for S232A neurons. Additionally, segmental velocity was affected only for retrograde vesicles in S232A axons, indicating potential reduction in dynein motor motility. Based on these differences in trafficking parameters, we hypothesize that decreased net velocity in S232D neurons is secondary to dramatic increase in pausing, while decreased net velocity in S232A neurons is related to reduced retrograde segmental velocity, which is more reflective of dynein motility. Consistent with this, net velocity impacts were more pronounced in the retrograde direction than in the anterograde direction.

**Fig. 7.**
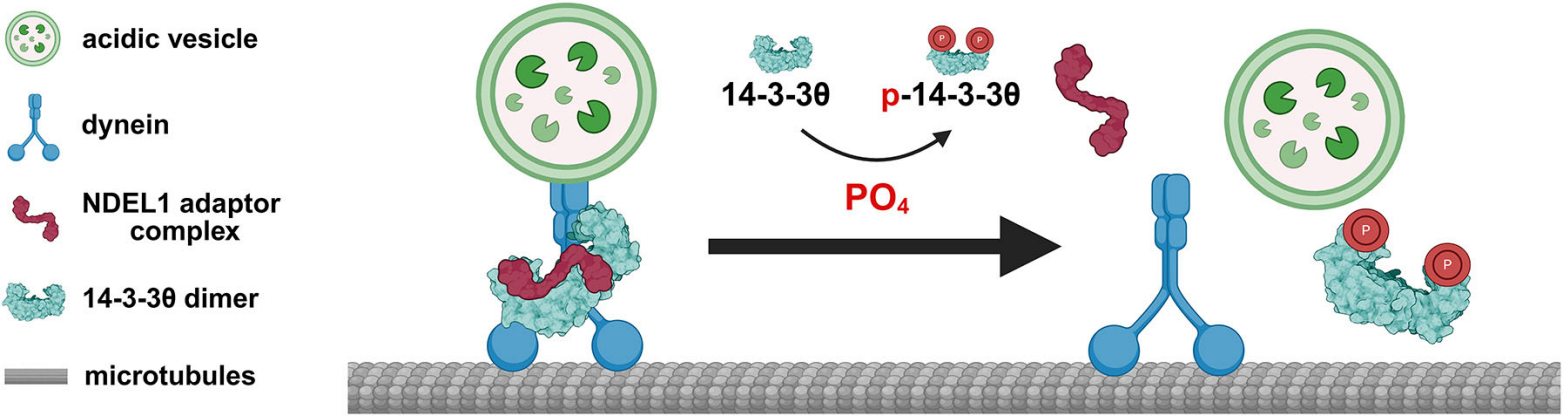
**Model of S232 phosphorylation effects on axonal trafficking.** Our data suggest a model in which 14-3-3θ phosphorylation acts as a signal to regulate motor protein-adaptor complex assembly and disassembly that allow vesicles to attach and de-attach from the motor tracks. In the absence of S232 phosphorylation, 14-3-3θ brings together dynein, NDEL1 and other parts of the motor complex to promote transport of acidic vesicles. Upon S232 phosphorylation, this complex is disassembled, and the vesicle can detach from the motor tracks. Either constant phosphorylation (modeled by S232D) or persistent lack of phosphorylation (modeled by S232A) cause dysfunctional transport due to the disruption of the normal cycling between motor complex assembly and disassembly. P, phospho. Created in BioRender by Yacoubian, T. A. (2025). https://BioRender.com/08flb9c. This figure was sublicensed under CC-BY 4.0 terms.

Overall, our AP-MS and trafficking data suggest a model in which 14-3-3θ phosphorylation at S232 serves as a regulatory signal for motor protein-adaptor complex assembly and disassembly that allow vesicles to hop on and off the motor tracks (Fig. 7). In the absence of S232 phosphorylation, 14-3-3θ brings together the trafficking complex, including dynein and NDEL1, to allow transport of acidic vesicles. Upon S232 phosphorylation, the complex is disassembled, and the vesicle is detached from the motor tracks. Being phosphorylated all the time (as mimicked by the S232D mutation) or never phosphorylated (as modeled by the S232A mutation) leads to dysfunctional transport due to the disruption of the normal cycling between motor complex assembly and disassembly. Our data showing alterations in vesicular transport observed in both S232A and S232D neurons are consistent with this interpretation.

The detailed molecular mechanisms by which S232 phosphorylation impacts acidic vesicle transport requires further investigation. The fact that we see deficits in both directions points to a possible mechanism involving bidirectional transport of cargo in axons. Many studies have shown that cargo often have dynein and kinesin motors attached simultaneously (Ma and Chisholm, 2002; Ligon et al., 2004; Hendricks et al., 2010). Paradoxically, inhibition of either kinesin or dynein negatively affects the other motor protein (Ally et al., 2009). Various trafficking models have suggested that the opposing motor protein will weakly associate with the microtubule and thus help the complex stay on the track and/or that motor proteins remain autoinhibited until direct binding of the opposing motor protein relieves this autoinhibition (Hancock, 2014). For example, the anterograde kinesin KIF1C promotes the retrograde transport of lysosomes involving a HOOK3-mediated adaptor complex that connects KIF1C to retrograde motor protein dynein, while KIF1C links HOOK3 to the lysosome cargo adaptor RUFY3 (Saji et al., 2024). The potential benefit of using adaptor proteins as connectors between motor proteins, as opposed to direct binding, is the increased flexibility, which allows opposing motor proteins to weakly engage microtubules and add additional tethering (Abid Ali et al., 2025). One possible explanation for the bidirectional deficits we see with the S232 mutants is that 14-3-3θ may serve as a scaffold for the formation of these bidirectional adaptor complexes. Phosphorylation at S232 could regulate the flexibility of the adapter complex. Ultimately, more experiments and biophysical models are required to further investigate the exact role 14-3-3θ plays in this transport machinery.

Because LysoTracker stains multiple acidic vesicles beyond lysosomes including some late endosomes and autophagosomes, further studies are needed to examine whether the transport of all or some of these vesicles is affected by S232 phosphorylation. Additionally, whether trafficking of other organelles is impacted by S232 phosphorylation requires further study. Evaluation of other functions associated with proteins binding differentially between S232A and S232D, including endocytosis and synaptic function, is warranted in the future. Of note, disrupted axonal transport of acidic vesicles, such as lysosomes, could affect the clearance of αsyn, the pathology of which initiates at axonal terminals in PD and DLB, in which increased S232 phosphorylation is observed (Schaser et al., 2020; Uchihara, 2017; Wong et al., 2019). Thus, disrupted lysosomal trafficking could be one key mechanism by which 14-3-3θ phosphorylation at S232 contributes to αsyn pathology in PD and DLB. Identification of kinases and/or phosphatases that regulate S232 phosphorylation could serve as potential targets to promote axonal trafficking in these disorders.

There are several limitations to the current study. We used an Emx1-Cre mouse line to knock in the S232 phosphorylation mutants into excitatory neurons in the cortex. However, because our cortical lysates include proteins from many cell types that express wild-type 14-3-3θ, the presence of wild-type 14-3-3θ may dilute out differences between S232D and S232A mutants. The antibody we used to perform these pulldowns does not distinguish between wild-type and mutant 14-3-3θ. Another limitation is that 14-3-3 PPIs tend to be transient with high specificity and low affinity (Wright and Dyson, 1999) so that certain transient 14-3-3θ PPIs could have been missed by our experimental approach. Of note, we did not detect certain known interactors, such as αsyn, in our AP-MS. These interactions may be transient and therefore not detected. Additionally, αsyn is difficult to detect at baseline due to trypsin digestion producing a bulky and highly charged C-terminal fragment. Our AP-MS approach also does not distinguish whether the interactor binds directly or indirectly through a complex to 14-3-3θ. Another limitation is that the S232D mutant does not perfectly mimic the serine phosphorylation, yet this is currently the most effective method for mimicking phosphorylation in *in vivo* mammalian systems. Advancement in genetic code expansion techniques may allow for more accurate mimicking of phosphorylation *in vivo* in the future (Qin and Liu, 2022). The S232D mutant also does not allow for any protein binding effects that may result from the actual movement of the C-terminal that occurs upon S232 phosphorylation (Pattanayak et al., 2024). Finally, our AP-MS was spread over two separate runs, in which we ran males and females separately; increased variability could be due to a batch effect or because the estrous cycle of females in a wide range of animals is known to affect protein expression and function (Unger et al., 2023; Shashikumar et al., 2018; Xin et al., 2023; Hojo and Kawato, 2018).

In conclusion, our study reveals that 14-3-3θ interactors in the brain include proteins important for neurodevelopment, neuron morphology and vesicle trafficking. 14-3-3θ phosphorylation impacts binding of 14-3-3θ to several proteins important in axonal trafficking and likely serves as a critical regulatory of axonal transport in neurons.

## MATERIALS AND METHODS

### Resources

Specific product IDs and vendors can be found in Tables S1 and S2.

### Mice

Mice were used following guidelines of the University of Alabama at Birmingham (UAB) Institutional Animal Care and Use Committee (IACUC) and the National Institutes of Health (NIH). Work with these animals was approved by UAB's IACUC. Conditional 14-3-3θ phospho-mutant (S232A or S232D) KI mice were crossed with an Emx1-Cre mouse line (Gorski et al., 2002) to express S232A or S232D 14-3-3θ mutant in the hippocampus and cortex, as previously described (Wang et al., 2025). Three-month-old mice were used for AP-MS studies.

### Immunoprecipitation for affinity purification

Samples were prepared and mass spectrometry run in two separate batches. The first batch of 3-month-old mice were all male and included four Cre+/− S232A+/+ (S232A) mice, four Cre+/− S232D+/+ (S232D) mice and four Cre+/− S232D−/− control mice. The second batch of 3-month-old mice were all female mice and contained four Cre+/− S232A+/+ (S232A) mice, four Cre+/− S232D+/+ (S232D) mice, four Cre+/− S232D−/− control mice and four Cre+/− S232A−/− control mice.

Cortices were collected and flash frozen and stored at −80°C until lysis. Brains were homogenized in 0.5% NP-40 lysis buffer (150 mM NaCl, 10 mM Tris-HCl, pH 7.4, 1 mM EGTA, 1 mM EDTA, 0.5% NP-40) with protease and phosphatase inhibitors added followed by sonication. Samples were centrifuged at 15,000 ***g*** for 60 min at 4°C. The supernatant was removed, and protein concentration was determined by bicinchoninic acid assay (BCA). 500 μg of protein was diluted in 200 μl lysis buffer for each immunoprecipitation (IP) replicate. Samples were incubated with antibody (Table S2) overnight on a rotator at 4°C. For each mouse cortical sample, four replicate pulldowns for 14-3-3θ and four replicate pulldowns for rabbit IgG were performed. Three replicates for 14-3-3θ pulldowns and three replicates for rabbit IgG control pulldowns were combined for mass spectrometry. The fourth replicate for 14-3-3θ and rabbit IgG control pulldowns were used to perform western blotting.

50 μl of dynabeads were washed twice with citrate-phosphate buffer (25 mM citric acid, 50 mM dibasic sodium phosphate dihydrate, pH 5.0) and then washed with PBS with 0.02% Tween 20 prior to being added to each IP reaction. After rotating at 4°C for 2 h, the sample-bead slurry was placed on a magnet on ice, and the supernatant was collected and saved as the post IP sample. The beads were then washed in PBS+0.02% Tween 20 five times. For western blotting, beads were resuspended in 15 μl 1× DTT loading buffer prior to boiling for 10 min at 95°C, and samples were then run on western blots.

### Mass spectrometry

Excess buffer was removed from each replicate sample, and they were reconstituted in 12 μl of 1× NuPAGE LDS sample buffer. Samples were eluted at 96°C for 10 min with shaking. Triple replicates were combined, reduced with DTT and denatured for 10 min at 70°C. Samples were run on a Novex NuPAGE 10% Bis-Tris Protein gel and separated at 200 V for 35 min. The gels were stained overnight with Novex Colloidal Blue Staining kit and destained. Each lane was cut into three separate molecular mass fractions and equilibrated in 100 mM ammonium bicarbonate. Each fraction was digested using Trypsin Gold (Mass Spectrometry Grade) overnight following the manufacturer's instructions. Digests were then diluted in 0.1% formic acid at 0.1 μg/μl.

### Nanoflow liquid chromatography-electrospray ionization-tandem mass spectrometry analysis and database searches

Digests were loaded onto a Thermo Orbitrap Velos Pro hybrid mass spectrometer, and data were processed as previously described to generate peptide identifications in SEQUEST (Mobley et al., 2023).

### AP-MS protein filtering and quality control

Peptide identifications were then filtered in Scaffold (Proteome Software). For filtering, we set cut-offs at minimum peptide length of five amino acids, no MH+1 charge states, peptide probabilities of greater than 80% confidence interval (c.i.), and at least two peptides per protein. The protein probabilities were set to a >95.0% c.i., resulting in an FDR of 0.4%.

Once proteins were identified, we wrote code in Python 3.6 to perform quality control (QC) checks on proteins to have high confidence in protein identification for general 14-3-3θ interactor analysis (Fig. 1B). First, our non-zero filter examined the number of samples that had zero spectral counts per group. We had 12 Cre control mice. A protein passed the non-zero filter if nine of the 12 samples had a non-zero spectral count. This reduced our starting list from 1465 to 252. We then filtered based on spectral count. The mean spectral count for each protein was taken across samples. If the average spectral count was ≥3, it passed our abundance test. This cut-off was determined as it was ∼1% of the average spectral count of 14-3-3θ in our samples. This reduced our list of proteins from 252 to 201. Proteins passing the abundance test were then compared to their IgG IP counterparts. To pass, a protein had to have ≥1.5-fold abundance in the 14-3-3θ pulldown compared to the IgG pulldown. This resulted in 175 proteins that passed all QC checks.

For analysis comparing S232A with S232D interactome, we included 12 Cre control, eight S232A and eight S232D mice. Testing was done on four males and four females for each phospho-mutant genotype. Four male S232D strain Cre control mice were used along with four female S232D strain Cre control and four male S232A Cre control mice. Peptides were identified the same as described above. This resulted in the identification of 1465 proteins. We again used a script in Python 3.6 to perform QC on identified proteins (Fig. 3B). First, we filtered out proteins with too many zero values. We performed a test on each of the three genotypes. Both sexes of a genotype must have at least three non-zero values each. If two of the three genotypes passed this test, a protein passed. This resulted in a list of 1465 proteins being reduced to 277. Proteins then had to have an average spectral count ≥3 in at least one of the three genotypes. This further filtered our list to 238 proteins. We once again filtered for IgG binding where two of the three genotypes must have ≥1.5-fold abundance in the 14-3-3θ pulldown compared to the IgG pulldown, with 208 proteins passing this filter. Finally, we performed a Wilcoxon rank-sum test using Python's scipy.stats library version 1.7.1 comparing S232A and S232D mice using a statistically significant cut-off of *P*≤0.05. 32 proteins passed our final statistical filter.

### Systems analysis

Systems analysis for general interactors and proteins showing a difference in binding to S232A versus S232D was run in Qiagen's IPA software (Krämer et al., 2014). Detailed analysis parameters can be found in the Supplementary Materials and Methods. Basic parameters were to include only experimentally observed datasets. When considering interacting/regulating molecules, ‘all available’ were selected, including biologic drug, protein, DNA, RNA, chemical, toxin and toxicant, among others. Data from all tissue types were considered, including immortalized cell lines, primary human/animal tissue, among others. A Fisher's exact test was used in these analyses to generate *P*-values for pathways, functions and upstream regulators. A Benjamin–Hochberg-corrected significance threshold was set for all *P*-values in these analyses. The exact way in which z-values are calculated can be found in Qiagen's initial IPA publication (Krämer et al., 2014). Heatmaps were generated in Qlucore Omics Explorer (Qlucore AB, Lund, Sweden). Samples in heatmaps underwent hierarchical clustering, and each protein was normalized between samples on a scale from −2 to 2. The volcano plot was generated in VolcaNoseR with a Wilcoxon rank-sum test *P*<0.05 and a |fold change|>1.5.

### Western blotting

Samples were loaded onto a 12% SDS-PAGE gel and transferred onto nitrocellulose membrane for 1 h in ice-cold transfer buffer (25 mM Tris, 190 mM glycine, 20% methanol, 0.1% SDS). Blots were then blocked in Intercept blocking buffer for 1 h at room temperature. Blots were then incubated in primary antibody (Table S2) in Intercept primary antibody diluent overnight at 4°C. After six 10-min washes in TBST (25 mM Tris-HCl pH 7.6, 137 mM NaCl, 0.1% Tween 20), blots were incubated in secondary antibodies diluted in Intercept primary antibody diluent+0.02% SDS for 1 h at room temperature. After six 10-min washes, blots for chemiluminescence were placed in ECL solution for 2 min and imaged on a Bio-Rad ChemiDoc Imaging System. For immunofluorescence, blots were imaged on an Odyssey CLX Licor machine.

### β-spectrin and dynamin 1 co-immunoprecipitations

Wild-type mouse cortex was homogenized in Pierce IP buffer (25 mM Tris-HCl pH 7.4, 150 mM NaCl, 1 mM EDTA, 1% NP40, 5% glycerol). For the β-spectrin IP, 500 µg protein lysate was incubated with 10 µg anti-β-spectrin antibody (Table S2) overnight alongside lysate incubated with rabbit IgG control. For dynamin 1, 1 mg protein was loaded with 10 µg anti-dynamin 1 antibody (Table S2) alongside rabbit IgG controls. After antibody-lysate incubation, dynabeads were added for 2 h rotating at 4°C. Beads were eluted with 25 µl 0.25 M acidic glycine buffer pH 3.0 for 25 min at room temperature. After elution, 4× DTT western loading buffer was added to elution samples, which were then boiled at 95°C for 10 min. Samples were run on 7.5% or 12% SDS-PAGE gel.

### Cell culture

SK-N-BE(2)-M17 cells, originally obtained from American Type Culture Collection, were developed to overexpress a V5-tagged 14-3-3θ construct with either an A or D mutation at S232 and validated via western blotting, as previously described (Slone et al., 2015). These stable cells did not exhibit signs of contamination. Cells were maintained in a 50/50 mixture of EMEM and F12-K medium supplemented with 10% fetal bovine serum (FBS) and 1% penicillin-streptomycin. Cells were treated with 500 μg/ml G418 sulfate to maintain selection for S232 phospho-mutant constructs.

### PLA

M17 cells expressing either V5-tagged S232A or D 14-3-3θ mutant were plated on 35 mm glass coverslips at 50,000 cells per well. 48 h later, cells were fixed in 4% paraformaldehyde for 20 min, blocked in 10% normal donkey serum (NDS) and 0.02% Triton X-100 in PBS, and incubated with primary antibodies (Table S2) diluted in PBS containing 1% NDS overnight at 4°C. PLA was performed according to the manufacturer's instructions. Cells were washed in PBS and treated with DuoLink PLUS and MINUS probes, followed by ligation, amplification and coverslipping. Cells were imaged on a confocal microscope (Nikon Eclipse Ti2 scanning confocal microscope) at 63×. Five random fields were taken on each coverslip, and images were quantified using Nikon NIS-Elements. Regions of interest (ROIs) were drawn around individual cells in focus across these five fields. The number of objects/area for each ROI was then normalized relative to the weighted average for that round. The PLA signal for a given condition was calculated as the average of all normalized number of objects/area across all ROIs for that condition.

### Primary neuronal cultures

Primary neurons were harvested, as described previously (Wang et al., 2018). Before hippocampal collection, postnatal day (P)0 pups were anesthetized on ice. Both hippocampi were collected and digested in papain for 30 min at 37°C. Hippocampi were mechanically homogenized and plated in Neurobasal A medium supplemented with B27, GlutaMax, penicillin-streptomycin and FBS. One day after plating, cells were treated with 1 μM cytarabine in medium containing no FBS to reduce glial proliferation.

### Axonal trafficking assay

Primary hippocampal neurons were plated on Xona dual chambers with 900 µm separation between chambers attached to a 55 mm Mattek dish. On DIV 10, medium was removed and saved as conditioned medium. 200 nM LysoTracker Red DND-99 was added to a 50%/50% mixture of conditioned and fresh supplemented Neurobasal A medium. The plates were incubated at 37°C for 1 h. After incubation, the medium was removed and replaced with a 50/50 mixture of conditioned and fresh media without LysoTracker. To assess axonal transport, plates were imaged at 63× with a Zeiss Observer high-speed live-cell imaging system with a Colibri2 cool LED 5-channel fast wavelength switching system and a Hamamatsu Orca Flash high-speed camera. Videos were recorded at the distal quarter of the 900 μm groove as only axons will reach that far into the chamber. One image was taken every 500 ms for 2 min (Movies 1, 2). Kymographs were generated and analyzed using KymoAnalyzer (Neumann et al., 2017), a freely available Fiji plugin. Note, we received an error on line 154 when running the v1.01 ‘Segments’ plugin as made on github. We changed the call to the ‘AssignSegments’ function on line 119 from ‘AssignSegments(Index[j]);’ to ‘AssignSegments(Index[j],j);’ to pass the j variable as a numeric identifier to distinguish segments. The ‘AssignSegments’ function was then altered to receive this numeric distinguisher due to an out of bounds index error on line 154. The original was ‘trackName=substring(Roi.getName,lengthOf(Roi.getName)-8,lengthOf(Roi.getName));’, which was changed to ‘trackName=Roi.getName+j;’.

### Statistical testing

AP-MS peptide identification, quantification, comparison testing and systems analysis were all performed as described above. In other experiments, statistical testing and graphing were performed in Prism v10.0.3. Groups were tested for normality using a Shapiro–Wilk test. Parametric groups underwent an unpaired two-tailed *t*-test. Non-parametric groups underwent a Mann–Whitney test. Four exceptions to this in the axonal trafficking data were the pause and switch counts in the S232D and S232A datasets. These datasets contain count data with an exceptional number of zeros. We performed a zero-inflated count model in SPSS, and histograms were generated in Prism. *P*-values and model coefficients reported are from the non-zero count model. Full statistical details and results are available in Dataset 3.

## Supplementary Material

10.1242/dmm.052405_sup1 Supplementary information

Dataset 1. Genera 1l4-3-3θ interactor mass spectrometry QC data.

Dataset 2. 14-3-3θ phosphorylation-specific interactor mass spectrometry QC data.

Dataset 3. Detailed statistical tests and analyses.

## Data and resource availability

The mass spectrometry proteomics data have been deposited to the ProteomeXchange Consortium via the PRIDE (Martens et al., 2005) partner repository with the dataset identifier PXD071112. All other relevant data and details of resources can be found within the article and its supplementary information.
