# Supplementary material for: 14-3-3θ phosphorylation at S232 reduces its interactome and regulates axonal trafficking
Source: Dis Model Mech. 2025 Dec 5;18(12):dmm052405. doi: 10.1242/dmm.052405 (PMC12714136; doi:10.1242/dmm.052405)
Supplement: Supplementary information [file dmm-18-052405-s1.pdf]

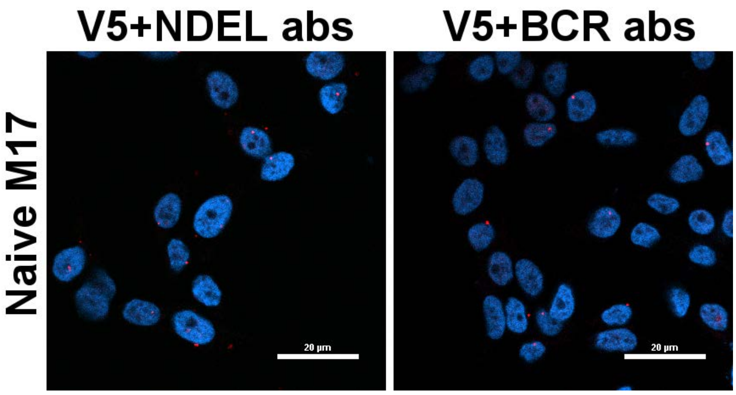

Fig. S1. Naive M17 cells lacking V5-tagged 14-3-3θ phospho-mutants lack strong PLA signal.

M17 cells not expressing V5-tagged 14-3-3θ phosphorylation mutant constructs show minimal PLA signal using V5 and NDEL1 antibodies as well as V5 and BCR antibodies.

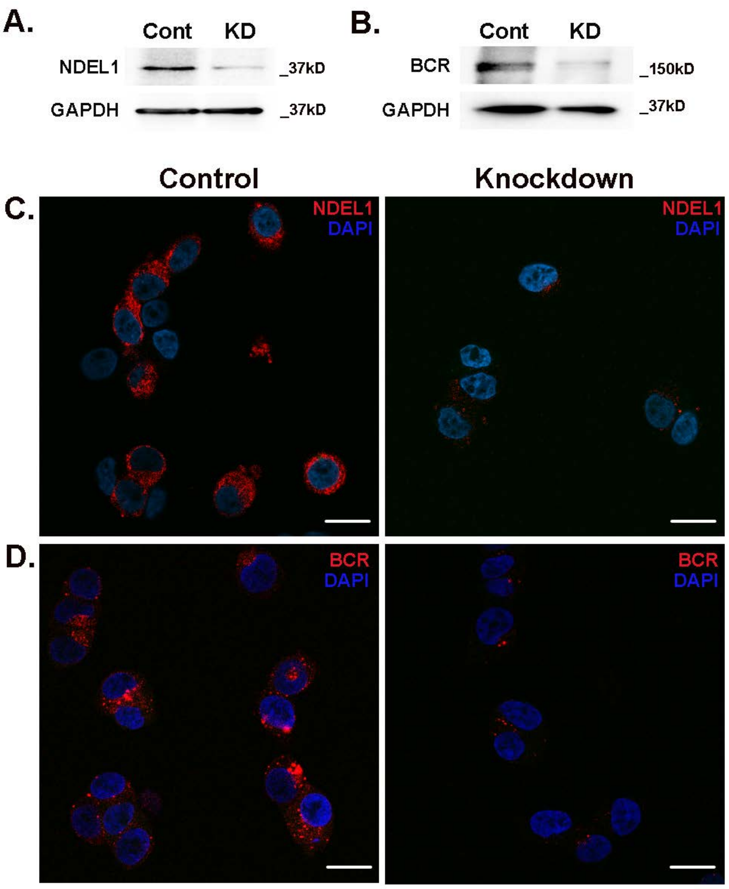

Fig. S2. Knockdown validation of antibodies against NDEL1 and BCR used for PLA.
A. Representative western blot of NDEL1 in M17 cells with non-target shRNA KD and M17 cells with shRNA-mediated KD against NDEL1.

B. Representative western blot of BCR in M17 cells with non-target shRNA KD and M17 cells with shRNA-mediated KD against BCR.

C. Representative immunostaining for NDEL1 (red) in control and M17 cells with shRNA-mediated KD against NDEL1. Blue is DAPI staining against nuclei. Scale bar = 10 µm.

D. Representative immunostaining for BCR (red) in control and M17 cells with shRNA-mediated KD against BCR. Blue is DAPI staining against nuclei. Scale bar = 10 µm.

Disease Models & Mechanisms • Supplementary information

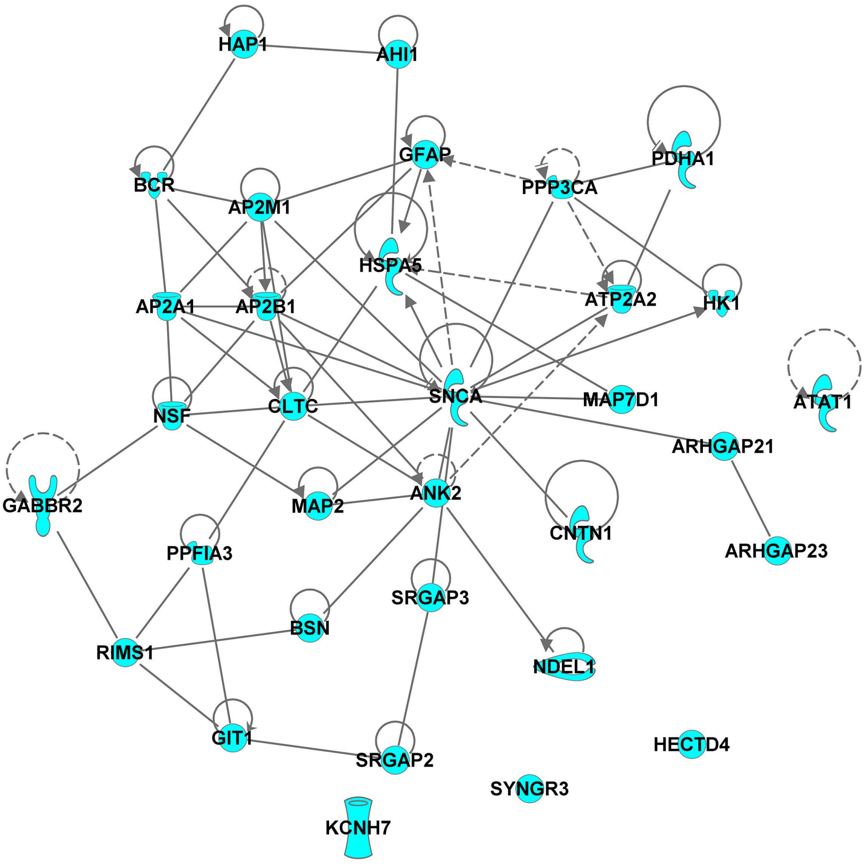

Fig. S3. 14-3-3θ interactors whose binding affinity are affected by S232 phosphorylation show dense interconnectedness with αsyn.

Our 32 phosphorylation-dependent binding proteins and αsyn were put into IPA's pathway-pathway generation tool and experimentally verified protein interactions were mapped between proteins showing a strong number of interactions between our proteins and αsynuclein.

Table S1. Materials and reagents.

Reagent	Company	Catalog number
**Protease Inhibitor**	Pierce	A32965
**Phosphatase inhibitor**	Pierce	A32957
**BCA Kit**	Pierce	23225
**Nitrocellulose 0.2 uM pore**	Amersham Protran	10600004
**PVDF IF 0.4 uM pore**	Immobilon	IPFL00010
**Intercept Blocking Buffer**	LICOR	927-60001
**Intercept Antibody Diluent**	LICOR	927-65001
**ECL Western Blotting Substrate**	Pierce	32106
**Super Signal Dura Extend Duration Substrate**	ThermoScientific	34076
**Duolink PLA Probe Anti-Rabbit PLUS**	Sigma-Aldrich	DUO92002
**Duolink PLA Probe Anti-Mouse MINUS**	Sigma-Aldrich	DUO92004
**Duolink In Situ Detection Reagents Red**	Sigma-Aldrich	DUO92008
**Prolong Diamond + DAPI**	ThermoFisher Scientific	P36962
**Protein A Dynabeads**	Invitrogen	10002D
**Protein G Dynabeads**	Invitrogen	1004D
**DynaMag™-2 Magnet**	Invitrogen	12321D
**Gibco Neurobasal-A Medium, minus phenol red**	Gibco	12349015
**Gibco Neurobasal Medium**	Gibco	21103049
**B27**	Gibco	12587001
**Papain**	Worthington Biochemical	3176
**Gibco CTS GlutaMAX-I Supplement**	Gibco	A1286001
**Fetal Bovine Serum**	Sigma	D9891
**Cytarabine CRS (Arac)**	LGC	EPC3350000
**35 mm Mattek Dish**	Mat-Tek	P35G-0-14-C
**50 mm Dish, No. 0 Coverslip, 30 mm Glass Diameter**	Mat-Tek	P50G-0-30-F
**Round Device 900 µm barrier 5PK dual chamber**	XONA	RD900
**Lysotracker Red DND-99**	Invitrogen	L7528
**F-12K Media**	ATCC	30-2004
**EMEM Media**	ATCC	30-2003
**Penicilin-Streptomycin**	Cytiva	SV30010
**Fetal Bovine Serum**	Sigma	D9891
**BCA Protein Assay Kit**	Thermo Fisher	PI23225
**NuPAGE LDS sample Buffer**	Invitrogen	NP0007
**Novex NuPAGE 10% Bis-Tris protein gel**	Invitrogen	NP0315BOX

Disease Models & Mechanisms • Supplementary information

**Novex Colloidal Blue Staining kit**	Invitrogen	LC6025
**Ammonium Bicarbonate**	Millipore SIGMA	A6141
**Trypsin Gold Mass Spectrometry Grade**	Promega	V5280
**100 micron I.D. x 13.5 cm pulled tip C-18 column (Jupiter 5um C18 300A Bulk Packing)**	Phenomenex	04A-4053
**100 micron I.D. x 13.5 cm pulled tip C-18 column (Molex capillary tubing 100umID)**	Fisher	50 110 8623

Table S2. Antibodies used.

Primary Antibody	Dilution	Source	Identifier	RRID
**14-3-3θ Rabbit polyclonal**	IP: 5ug	Bethyl	A303-146A	AB_10894703
**14-3-3 theta mouse monoclonal (3B9)**	Western: 1:200	Santa Cruz	sc-59414	AB_2218234
**14-3-3 theta mouse monoclonal (5J20)**	PLA: 1:250	Santa Cruz	sc-69720	AB_2218224
**Beta Spectrin 1 Rabbit polyclonal**	IP: 5ug<br>Western: 1:1000<br>PLA: 1:250	Bethyl	A300-936A	AB_2194508
**Dynamin 1 Rabbit polyclonal**	IP: 10 ug<br>Western: 1:1000<br>PLA: 1:250	Thermo-Fisher	PA1-660	AB_325845
**NDEL1 Rabbit polyclonal**	PLA: 1:250	ProteinTech	17262-1-AP	AB_2235821
**BCR Rabbit polyclonal**	PLA: 1:250	Bethyl	A302-057A	AB_1604260
**Goat anti-Rabbit Light Chain Antibody HRP Conjugated**	Western 1:5000	Bethyl	A120-113P	AB_10755117
**Goat anti-Mouse IgG-Fc Fragment Antibody HRP Conjugated**	Western 1:5000	Bethyl	A90-131P	AB_67175
**IRDye 680RD Goat anti-Mouse IgG Secondary Antibody**	Western 1:5000	Licor	926-68070	AB_10956588

Dataset 1. General 14-3-3θ interactor mass spectrometry QC data.

Available for download at
https://journals.biologists.com/dmm/article-lookup/doi/10.1242/dmm.052405#supplementary-data

Dataset 2. 14-3-3θ phosphorylation-specific interactor mass spectrometry QC data.

Available for download at
https://journals.biologists.com/dmm/article-lookup/doi/10.1242/dmm.052405#supplementary-data

Dataset 3. Detailed statistical tests and analyses.

Available for download at

https://journals.biologists.com/dmm/article-lookup/doi/10.1242/dmm.052405#supplementary-data

Disease Models & Mechanisms • Supplementary information

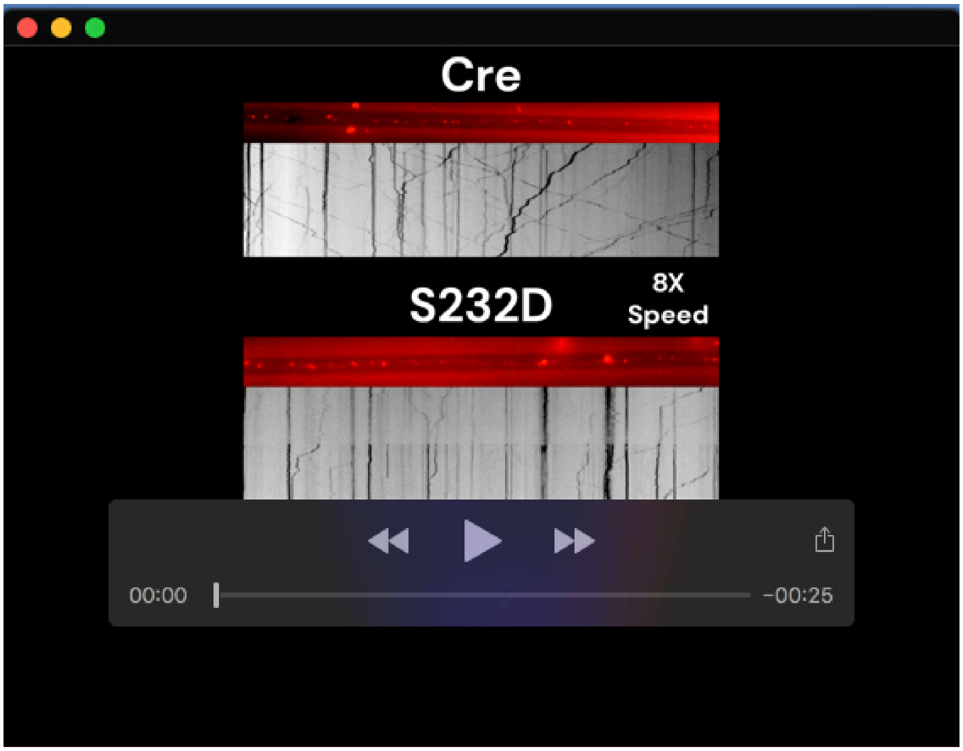

Movie 1. Live cell imaging videos of representative S232D and Cre control LysoTracker stained axons and their respective kymographs.

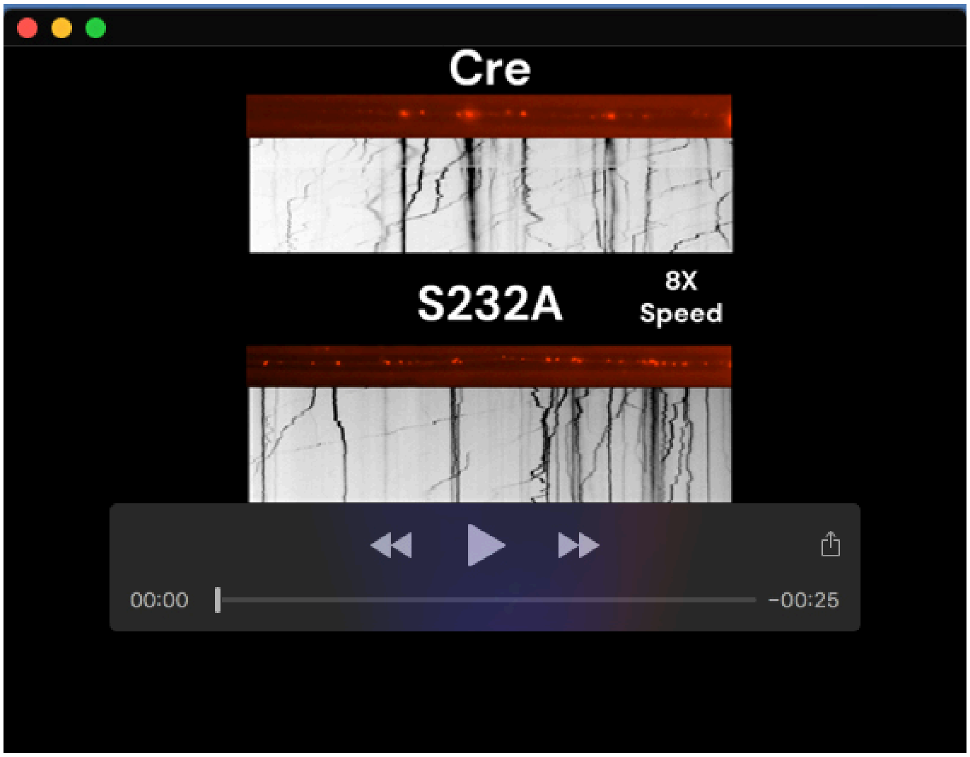

Movie 2. Live cell imaging videos of representative S232A and Cre control LysoTracker stained axons and their respective kymographs.

Disease Models & Mechanisms • Supplementary information

Supplementary Materials and Methods

INGENUITY
PATHWAY ANALYSIS

Analysis Name: General Binding Mean SC - 2024-01-25 10:57 AM
Analysis Creation Date: 2024-01-25
Build version: exported
Content version: 107193442 (Release Date: 2023-11-18)

Experiment Metadata

Name	Value

Analysis Settings

Reference set: Ingenuity Knowledge Base (Genes Only)
Relationship to include: Direct and Indirect
Includes Endogenous Chemicals
Optional Analyses: My Pathways My List

Filter Summary:
Consider only molecules and/or relationships where
(species = Mouse OR Uncategorized OR Human OR Rat) AND
(confidence = Experimentally Observed) AND
(tissues/cell lines = Smooth Muscle OR Heart OR Macrophages not otherwise specified OR Gray Matter OR Other Lymphoma Cell Lines OR Testis OR Bladder OR Other Cell Line OR J-774A.1 OR Pro-B lymphocytes OR Dorsal Root Ganglion OR Other Melanoma Cell Lines OR Thymus OR Kidney OR HepG2 OR OVCAR-8 OR OVCAR-4 OR Monocyte-derived macrophage OR Other Macrophages OR Epithelial cells not

(c) 2000-2024 QIAGEN. All rights reserved.

Disease Models & Mechanisms • Supplementary information

otherwise specified OR Naive B cells OR TK-10 OR Peripheral blood leukocytes not otherwise specified OR Adipocytes OR SN12C OR B lymphocytes not otherwise specified OR U251 OR Mammary Gland OR Mast cells OR Calvaria OR Kidney Cancer Cell Lines not otherwise specified OR Adrenal Gland OR Parietal Lobe OR Activated Vd2 Gamma-delta T cells OR RAW 264.7 OR BT-549 OR Cos-7 cells OR MDA-N OR Hippocampus OR LOX IMVI OR SF-295 OR Skin OR A549-ATCC OR HS 578T OR BDCA-1+ dendritic cells OR Natural T-regulatory cells OR PBMCs OR NB4 OR Crypt OR 3T3-L1 cells OR Putamen OR WEHI-231 OR Cartilage Tissue OR CD4+ T-lymphocytes OR Brain OR Thyroid Gland OR CAKI-1 OR Th17 cells OR Other Breast Cancer Cell Lines OR Lung OR Cerebellum OR Other CNS Cell Lines OR Other NK cells OR Jurkat OR Oocytes OR HOP-62 OR SK-MEL-2 OR Trachea OR PC-3 OR Cortical neurons OR SF-268 OR NCI-H332M OR Other Colon Cancer Cell Lines OR HT29 OR Lymphoma Cell Lines not otherwise specified OR Swiss 3T3 cells OR Bone marrow-derived macrophages OR SW-480 OR Vascular smooth muscle cells OR CNS Cell Lines not otherwise specified OR PANC-1 OR Pituitary Gland OR Other Fibroblast cell lines OR HuH7 OR Other Stem cells OR Ovarian Cancer Cell Lines not otherwise specified OR Megakaryocytes OR Epidermis OR Immune cells not otherwise specified OR Retina OR Other Lung Cancer Cell Lines OR Caudate Nucleus OR Other Memory T lymphocytes OR SR OR Other Leukemia Cell Lines OR Other Teratocarcinoma Cell Lines OR IGROV1 OR Nucleus Accumbens OR Osteoblasts OR HOP-92 OR Myeloid dendritic cells OR Plasmacytoid dendritic cells OR NCI-H522 OR Lymphocytes not otherwise specified OR Pyramidal neurons OR OVCAR-5 OR Other Neuroblastoma Cell Lines OR Effector memory RA+ cytotoxic T cells OR HEL OR Other Mononuclear leukocytes OR Osteosarcoma Cell Lines not otherwise specified OR MALME-3M OR Purkinje cells OR INS-1 OR HCT-116 OR Granulocytes not otherwise specified OR Langerhans cells OR Mature monocyte-derived dendritic cells OR Effector T cells OR Memory B cells OR Pancreatic Cancer Cell Lines not otherwise specified OR Trigeminal Ganglion OR Other Hepatoma Cell Lines OR Substantia Nigra OR Other Myeloma Cell Lines OR Dermis OR Teratocarcinoma Cell Lines not otherwise specified OR Cornea OR Large Intestine OR A375 OR Mesenchymal stem cells OR K-562 OR SW-620 OR Activated CD56bright NK cells OR Pancreas OR Other Immune cells OR Other Dendritic cells OR OVCAR-3 OR Microglia OR Other Granulocytes OR RBL-2H3 OR Th1 cells OR Blood platelets OR UO-31 OR Other Monocytes OR Activated helper T cells OR SF-539 OR Adipose OR Effector memory cytotoxic T cells OR H460 OR Other Organ Systems OR Corpus Callosum OR Cerebral Cortex OR Cerebral Ventricles OR Peripheral blood lymphocytes OR SK-MEL-28 OR Placenta OR Hepatoma Cell Lines not otherwise specified OR PC-12 cells OR U87MG OR HMC-1 OR Fibroblasts OR Monocytes not otherwise specified OR MDA-MB-231 OR CD56dim NK cells OR T47-D OR Cells not otherwise specified OR Subventricular Zone OR Amygdala OR Other Macrophage Cancer Cell Lines OR Peritoneal macrophages OR CCRF-CEM OR Cervical cancer cell line not otherwise specified OR NIH/3T3 cells OR Forestomach OR Leukemia Cell Lines not otherwise specified OR Plasma cells OR Other Cervical cancer cell line OR Neutrophils OR Other Neurons OR Th2 cells OR Vd2 Gamma-delta T cells OR HCC-2998 OR Endothelial cells not otherwise specified OR Murine NKT cells OR HL-60 OR HeLa OR Activated Vd1 Gamma-delta T cells OR Stromal cells OR Striatum OR Hep3B OR Activated CD56dim NK cells OR Cardiomyocytes OR KM-12 OR Other Immune cell lines OR Prostate Cancer Cell Lines not otherwise specified OR Peripheral blood monocytes OR Other Osteosarcoma

(c) 2000-2024 QIAGEN. All rights reserved.

Disease Models & Mechanisms • Supplementary information

Cell Lines OR CD34+ cells OR Other Nervous System OR Immune cell lines not otherwise specified OR LNCaP cells OR Spleen OR Stomach OR Thalamus OR Lymph node OR DU-145 OR Other Tissues and Primary Cells OR THP-1 OR U266 OR Kidney cell lines not otherwise specified OR Dendritic cells not otherwise specified OR ACHN OR M14 OR Ovary OR Melanoma Cell Lines not otherwise specified OR Pre-B lymphocytes OR Keratinocytes OR Other B lymphocytes OR Olfactory Bulb OR Thymocytes OR Other Endothelial cells OR NCI-H226 OR Memory T lymphocytes not otherwise specified OR Esophagus OR Central memory helper T cells OR Other Lymphocytes OR BA/F3 OR Other Epithelial cells OR A498 OR Microvascular endothelial cells OR Other Pheochromocytoma cell lines OR MDA-MB-361 OR Neuroblastoma Cell Lines not otherwise specified OR Effector memory helper T cells OR Sertoli cells OR Fibroblast cell lines not otherwise specified OR Other Kidney Cancer Cell Lines OR U2OS OR Other Smooth muscle cells OR Prostate Gland OR Cell Line not otherwise specified OR Monocyte-derived dendritic cells not otherwise specified OR Vd1 Gamma-delta T cells OR 293 cells OR Bone marrow cells not otherwise specified OR Macrophage Cancer Cell Lines not otherwise specified OR Naive helper T cells OR J774 OR MDA-MB-435 OR Splenocytes OR Lens OR MDA-MB-468 OR T lymphocytes not otherwise specified OR Embryonic stem cells OR MG-63 OR Granulosa cells OR NT2/D1 OR UACC-62 OR Melanocytes OR Hypothalamus OR Stem cells not otherwise specified OR Ventricular Zone OR SK-OV-3 OR HUVEC cells OR Granule Cell Layer OR NK cells not otherwise specified OR Caco2 cells OR Mononuclear leukocytes not otherwise specified OR NCI-ADR-RES OR MCF7 OR Granule cells OR Uterus OR UACC-257 OR Medulla Oblongata OR CD56bright NK cells OR Other Kidney cell lines OR Nervous System not otherwise specified OR Colon Cancer Cell Lines not otherwise specified OR Cytotoxic T cells OR Beta islet cells OR MEF cells OR Myeloma Cell Lines not otherwise specified OR Other Ovarian Cancer Cell Lines OR Eosinophils OR Other T lymphocytes OR Breast Cancer Cell Lines not otherwise specified OR Sciatic Nerve OR COLO205 OR Other Peripheral blood leukocytes OR Liver OR Hepatocytes OR Smooth muscle cells not otherwise specified OR White Matter OR Pheochromocytoma cell lines not otherwise specified OR Skeletal Muscle OR HCT-15 OR RKO OR U937 OR EKVX OR MOLT-4 OR SNB-75 OR Immature monocyte-derived dendritic cells OR Other Pancreatic Cancer Cell Lines OR Organ Systems not otherwise specified OR Lung Cancer Cell Lines not otherwise specified OR Spinal Cord OR RXF-393 OR BDCA-3+ dendritic cells OR Chondrocytes OR 786-0 OR Choroid Plexus OR Tissues and Primary Cells not otherwise specified OR Hematopoietic progenitor cells OR Salivary Gland OR Astrocytes OR Other Prostate Cancer Cell Lines OR P19 OR RPMI-8266 OR Brainstem OR Intraepithelial T lymphocytes OR Central memory cytotoxic T cells OR Other Monocyte-derived dendritic cells OR Small Intestine OR Bone marrow-derived dendritic cells OR Min6 OR SK-N-SH OR Other Bone marrow cells OR A2780 OR Other Cells OR Neurons not otherwise specified OR BT-474 OR NCI-H23 OR SK-MEL-5) AND

(mol. types = biologic drug OR canonical pathway OR chemical - endogenous mammalian OR chemical - endogenous non-mammalian OR chemical - kinase inhibitor OR chemical - other OR chemical - protease inhibitor OR chemical drug OR chemical reagent OR chemical toxicant OR complex OR cytokine OR disease OR enzyme OR function OR fusion gene/product OR G-protein coupled receptor OR group OR growth factor OR ion channel OR kinase OR ligand-dependent nuclear receptor OR mature microRNA OR microRNA OR other OR peptidase OR

(c) 2000-2024 QIAGEN. All rights reserved.

Disease Models & Mechanisms • Supplementary information

Summary of Analysis - General Binding Mean SC - 2024-01-25 10:57 AM

phosphatase OR transcription regulator OR translation regulator OR transmembrane receptor OR transporter) AND (data sources = An Open Access Database of Genome-wide Association Results OR BIND OR BioGRID OR Catalogue Of Somatic Mutations In Cancer (COSMIC) OR Chemical Carcinogenesis Research Information System (CCRIS) OR Clinical Genome Resource (ClinGen) OR ClinicalTrials.gov OR ClinVar OR Cognia OR DIP OR DrugBank OR Gene Ontology (GO) OR GVK Biosciences OR Hazardous Substances Data Bank (HSDB) OR HumanCyc OR Ingenuity Expert Findings OR Ingenuity ExpertAssist Findings OR IntAct OR Interactome studies OR MIPS OR miRBase OR miRecords OR Mouse Genome Database (MGD) OR Obesity Gene Map Database OR Online Mendelian Inheritance in Man (OMIM) OR Reactome OR TarBase OR TargetScan Human OR TargetScan Mouse)

Top Canonical Pathways

Name	p-value	Overlap
**L1CAM interactions**	3.18E-20	15.2 % 19/125
**Glutamate binding, activation of AMPA receptors and synaptic plasticity**	1.98E-18	37.5 % 12/32
**Activation of NMDA receptors and postsynaptic events**	2.63E-14	15.5 % 13/84
**Assembly and cell surface presentation of NMDA receptors**	3.56E-13	23.3 % 10/43
**Translocation of SLC2A4 (GLUT4) to the plasma membrane**	2.68E-12	15.5 % 11/71

Top Upstream Regulators

Upstream Regulators

(c) 2000-2024 QIAGEN. All rights reserved.

INGENUITY PATHWAY ANALYSIS

Disease Models & Mechanisms • Supplementary information

Summary of Analysis - General Binding Mean SC - 2024-01-25 10:57 AM

Name	p-value	Predicted Activation
**MAPT**	1.77E-47	
**PSEN1**	1.21E-40	
**APP**	4.00E-35	
**HTT**	2.39E-21	
**sodium tungstate**	1.16E-16	

Causal Network

Name	p-value	Predicted Activation
**anisomycin**	5.66E-21	
**TAF1**	6.21E-20	
**RPL11**	1.25E-18	
**NCL**	1.30E-18	
**CDK11**	9.01E-18	

Top Diseases and Bio Functions

Diseases and Disorders

Name	p-value range	# Molecules
**Hereditary Disorder**	5.61E-05 - 6.18E-32	104
**Neurological Disease**	5.78E-05 - 6.18E-32	156
**Organismal Injury and Abnormalities**	5.84E-05 - 6.18E-32	166
**Skeletal and Muscular Disorders**	5.61E-05 - 6.96E-26	90

(c) 2000-2024 QIAGEN. All rights reserved.

INGENUITY PATHWAY ANALYSIS

Summary of Analysis - General Binding Mean SC - 2024-01-25 10:57 AM

**Psychological Disorders**	4.92E-05 - 3.93E-25	88

Molecular and Cellular Functions

Name	p-value range	# Molecules
**Cell Morphology**	5.36E-05 - 8.93E-42	96
**Cellular Development**	4.07E-05 - 8.93E-42	107
**Cellular Growth and Proliferation**	4.07E-05 - 8.93E-42	105
**Cellular Assembly and Organization**	5.41E-05 - 5.00E-41	116
**Cellular Function and Maintenance**	5.41E-05 - 5.00E-41	126

Physiological System Development and Function

Name	p-value range	# Molecules
**Nervous System Development and Function**	5.41E-05 - 8.93E-42	108
**Organismal Development**	5.41E-05 - 8.93E-42	114
**Tissue Development**	5.41E-05 - 8.93E-42	98
**Tissue Morphology**	4.04E-05 - 1.11E-31	82
**Embryonic Development**	5.41E-05 - 3.24E-23	78

Top Tox Functions

Assays: Clinical Chemistry and Hematology

(c) 2000-2024 QIAGEN. All rights reserved.

INGENUITY PATHWAY ANALYSIS
Disease Models & Mechanisms • Supplementary information

Summary of Analysis - General Binding Mean SC - 2024-01-25 10:57 AM

Name	p-value range	# Molecules
**Increased Levels of Potassium**	2.10E-02 - 2.10E-02	1
**Increased Levels of Hematocrit**	1.66E-01 - 1.66E-01	2

Cardiotoxicity

Name	p-value range	# Molecules
**Heart Failure**	7.47E-02 - 1.18E-06	15
**Cardiac Dysfunction**	1.16E-01 - 8.82E-06	16
**Congenital Heart Anomaly**	1.68E-01 - 2.46E-05	13
**Cardiac Arrythmia**	4.36E-01 - 7.01E-05	12
**Cardiac Enlargement**	2.69E-01 - 5.08E-04	17

Hepatotoxicity

Name	p-value range	# Molecules
**Liver Hyperplasia/Hyperproliferation**	3.77E-01 - 4.54E-12	102
**Hepatocellular carcinoma**	1.85E-01 - 1.79E-06	41
**Liver Proliferation**	1.74E-01 - 1.40E-02	5
**Liver Fibrosis**	3.07E-01 - 1.43E-02	6
**Liver Inflammation/Hepatitis**	5.11E-01 - 1.65E-02	6

Nephrotoxicity

(c) 2000-2024 QIAGEN. All rights reserved.

INGENUITY PATHWAY ANALYSIS

Summary of Analysis - General Binding Mean SC - 2024-01-25 10:57 AM

Name	p-value range	# Molecules
**Renal Necrosis/Cell Death**	2.69E-01 - 1.16E-03	12
**Kidney Failure**	4.57E-01 - 2.13E-03	3
**Renal Damage**	1.39E-01 - 2.13E-03	6
**Nephrosis**	2.51E-01 - 4.84E-03	6
**Glomerular Injury**	1.00E00 - 7.03E-03	8

Top Regulator Effect Networks

Top Networks

ID	Associated Network Functions	Score
**1**	Nervous System Development and Function, Organ Morphology, Tissue Morphology	40
**2**	Cellular Development, Cellular Growth and Proliferation, Nervous System Development and Function	32
**3**	Auditory Disease, Auditory and Vestibular System Development and Function, Organ Morphology	30

(c) 2000-2024 QIAGEN. All rights reserved.

INGENUITY PATHWAY ANALYSIS
Disease Models & Mechanisms • Supplementary information

Summary of Analysis - General Binding Mean SC - 2024-01-25 10:57 AM

4	Cell-To-Cell Signaling and Interaction, Nervous System Development and Function, Cellular Function and Maintenance	23
5	Hematological Disease, Hereditary Disorder, Organismal Injury and Abnormalities	23

Top Tox Lists

Name	p-value	Overlap
Mitochondrial Dysfunction	4.86E-09	4.5 % 16/355
Cell Cycle: G2/M DNA Damage Checkpoint Regulation	3.55E-05	9.4 % 5/53
Renal Inorganic Phosphate Homeostasis (Mouse)	7.24E-04	33.3 % 2/6
Renal Necrosis/Cell Death	3.19E-03	1.8 % 12/679
Cardiac Necrosis/Cell Death	3.63E-03	2.3 % 8/353

Top My Lists

Name	p-value	Overlap
1.5 fold cutoff A VS D	8.67E-101	90.6 % 48/53

(c) 2000-2024 QIAGEN. All rights reserved.

INGENUITY PATHWAY ANALYSIS

Disease Models & Mechanisms • Supplementary information

Summary of Analysis - General Binding Mean SC - 2024-01-25 10:57 AM

Top My Pathways

Top ML Disease Pathways

Name	p-value	Overlap
Paroxysmal movement disorder	4.35E-08	14.6 % 7/48
Infection by Dengue virus 2	1.07E-06	12.5 % 6/48
Rectum tumor	3.32E-06	10.3 % 6/58
Rectosigmoid neoplasm	3.32E-06	10.3 % 6/58
Axonal degeneration	1.12E-05	11.9 % 5/42

Top Analysis-Ready Molecules

Molecules	Expr. Value	Chart
YWHAQ	481.833	
YWHAZ	353.586	
YWHAB	237.100	
SRCIN1	164.852	
SPTAN1	128.703	
ACTB	126.632	
CAMK2A	124.593	
YWHAH	110.305	
TUBB3	103.801	
MYH10	102.920	

(c) 2000-2024 QIAGEN. All rights reserved.

INGENUITY PATHWAY ANALYSIS

INGENUITY
PATHWAY ANALYSIS

Analysis Name: A vs D IPA 1-25-24 - 2024-01-25 11:26 AM
Analysis Creation Date: 2024-01-25
Build version: exported
Content version: 107193442 (Release Date: 2023-11-18)

Experiment Metadata

Name	Value

Analysis Settings

Reference set: Ingenuity Knowledge Base (Genes Only)
Relationship to include: Direct and Indirect
Includes Endogenous Chemicals
Optional Analyses: My Pathways My List

Filter Summary:
Consider only molecules and/or relationships where
(species = Mouse OR Uncategorized OR Human OR Rat) AND
(confidence = Experimentally Observed) AND
(tissues/cell lines = Smooth Muscle OR Heart OR Macrophages not otherwise specified OR Gray Matter OR Other Lymphoma Cell Lines OR Testis OR Bladder OR Other Cell Line OR J-774A.1 OR Pro-B lymphocytes OR Dorsal Root Ganglion OR Other Melanoma Cell Lines OR Thymus OR Kidney OR HepG2 OR OVCAR-8 OR OVCAR-4 OR Monocyte-derived macrophage OR Other Macrophages OR Epithelial cells not

(c) 2000-2024 QIAGEN. All rights reserved.

Disease Models & Mechanisms • Supplementary information

otherwise specified OR Naive B cells OR TK-10 OR Peripheral blood leukocytes not otherwise specified OR Adipocytes OR SN12C OR B lymphocytes not otherwise specified OR U251 OR Mammary Gland OR Mast cells OR Calvaria OR Kidney Cancer Cell Lines not otherwise specified OR Adrenal Gland OR Parietal Lobe OR Activated Vd2 Gamma-delta T cells OR RAW 264.7 OR BT-549 OR Cos-7 cells OR MDA-N OR Hippocampus OR LOX IMVI OR SF-295 OR Skin OR A549-ATCC OR HS 578T OR BDCA-1+ dendritic cells OR Natural T-regulatory cells OR PBMCs OR NB4 OR Crypt OR 3T3-L1 cells OR Putamen OR WEHI-231 OR Cartilage Tissue OR CD4+ T-lymphocytes OR Brain OR Thyroid Gland OR CAKI-1 OR Th17 cells OR Other Breast Cancer Cell Lines OR Lung OR Cerebellum OR Other CNS Cell Lines OR Other NK cells OR Jurkat OR Oocytes OR HOP-62 OR SK-MEL-2 OR Trachea OR PC-3 OR Cortical neurons OR SF-268 OR NCI-H332M OR Other Colon Cancer Cell Lines OR HT29 OR Lymphoma Cell Lines not otherwise specified OR Swiss 3T3 cells OR Bone marrow-derived macrophages OR SW-480 OR Vascular smooth muscle cells OR CNS Cell Lines not otherwise specified OR PANC-1 OR Pituitary Gland OR Other Fibroblast cell lines OR HuH7 OR Other Stem cells OR Ovarian Cancer Cell Lines not otherwise specified OR Megakaryocytes OR Epidermis OR Immune cells not otherwise specified OR Retina OR Other Lung Cancer Cell Lines OR Caudate Nucleus OR Other Memory T lymphocytes OR SR OR Other Leukemia Cell Lines OR Other Teratocarcinoma Cell Lines OR IGROV1 OR Nucleus Accumbens OR Osteoblasts OR HOP-92 OR Myeloid dendritic cells OR Plasmacytoid dendritic cells OR NCI-H522 OR Lymphocytes not otherwise specified OR Pyramidal neurons OR OVCAR-5 OR Other Neuroblastoma Cell Lines OR Effector memory RA+ cytotoxic T cells OR HEL OR Other Mononuclear leukocytes OR Osteosarcoma Cell Lines not otherwise specified OR MALME-3M OR Purkinje cells OR INS-1 OR HCT-116 OR Granulocytes not otherwise specified OR Langerhans cells OR Mature monocyte-derived dendritic cells OR Effector T cells OR Memory B cells OR Pancreatic Cancer Cell Lines not otherwise specified OR Trigeminal Ganglion OR Other Hepatoma Cell Lines OR Substantia Nigra OR Other Myeloma Cell Lines OR Dermis OR Teratocarcinoma Cell Lines not otherwise specified OR Cornea OR Large Intestine OR A375 OR Mesenchymal stem cells OR K-562 OR SW-620 OR Activated CD56bright NK cells OR Pancreas OR Other Immune cells OR Other Dendritic cells OR OVCAR-3 OR Microglia OR Other Granulocytes OR RBL-2H3 OR Th1 cells OR Blood platelets OR UO-31 OR Other Monocytes OR Activated helper T cells OR SF-539 OR Adipose OR Effector memory cytotoxic T cells OR H460 OR Other Organ Systems OR Corpus Callosum OR Cerebral Cortex OR Cerebral Ventricles OR Peripheral blood lymphocytes OR SK-MEL-28 OR Placenta OR Hepatoma Cell Lines not otherwise specified OR PC-12 cells OR U87MG OR HMC-1 OR Fibroblasts OR Monocytes not otherwise specified OR MDA-MB-231 OR CD56dim NK cells OR T47-D OR Cells not otherwise specified OR Subventricular Zone OR Amygdala OR Other Macrophage Cancer Cell Lines OR Peritoneal macrophages OR CCRF-CEM OR Cervical cancer cell line not otherwise specified OR NIH/3T3 cells OR Forestomach OR Leukemia Cell Lines not otherwise specified OR Plasma cells OR Other Cervical cancer cell line OR Neutrophils OR Other Neurons OR Th2 cells OR Vd2 Gamma-delta T cells OR HCC-2998 OR Endothelial cells not otherwise specified OR Murine NKT cells OR HL-60 OR HeLa OR Activated Vd1 Gamma-delta T cells OR Stromal cells OR Striatum OR Hep3B OR Activated CD56dim NK cells OR Cardiomyocytes OR KM-12 OR Other Immune cell lines OR Prostate Cancer Cell Lines not otherwise specified OR Peripheral blood monocytes OR Other Osteosarcoma

(c) 2000-2024 QIAGEN. All rights reserved.

Disease Models & Mechanisms • Supplementary information

Cell Lines OR CD34+ cells OR Other Nervous System OR Immune cell lines not otherwise specified OR LNCaP cells OR Spleen OR Stomach OR Thalamus OR Lymph node OR DU-145 OR Other Tissues and Primary Cells OR THP-1 OR U266 OR Kidney cell lines not otherwise specified OR Dendritic cells not otherwise specified OR ACHN OR M14 OR Ovary OR Melanoma Cell Lines not otherwise specified OR Pre-B lymphocytes OR Keratinocytes OR Other B lymphocytes OR Olfactory Bulb OR Thymocytes OR Other Endothelial cells OR NCI-H226 OR Memory T lymphocytes not otherwise specified OR Esophagus OR Central memory helper T cells OR Other Lymphocytes OR BA/F3 OR Other Epithelial cells OR A498 OR Microvascular endothelial cells OR Other Pheochromocytoma cell lines OR MDA-MB-361 OR Neuroblastoma Cell Lines not otherwise specified OR Effector memory helper T cells OR Sertoli cells OR Fibroblast cell lines not otherwise specified OR Other Kidney Cancer Cell Lines OR U2OS OR Other Smooth muscle cells OR Prostate Gland OR Cell Line not otherwise specified OR Monocyte-derived dendritic cells not otherwise specified OR Vd1 Gamma-delta T cells OR 293 cells OR Bone marrow cells not otherwise specified OR Macrophage Cancer Cell Lines not otherwise specified OR Naive helper T cells OR J774 OR MDA-MB-435 OR Splenocytes OR Lens OR MDA-MB-468 OR T lymphocytes not otherwise specified OR Embryonic stem cells OR MG-63 OR Granulosa cells OR NT2/D1 OR UACC-62 OR Melanocytes OR Hypothalamus OR Stem cells not otherwise specified OR Ventricular Zone OR SK-OV-3 OR HUVEC cells OR Granule Cell Layer OR NK cells not otherwise specified OR Caco2 cells OR Mononuclear leukocytes not otherwise specified OR NCI-ADR-RES OR MCF7 OR Granule cells OR Uterus OR UACC-257 OR Medulla Oblongata OR CD56bright NK cells OR Other Kidney cell lines OR Nervous System not otherwise specified OR Colon Cancer Cell Lines not otherwise specified OR Cytotoxic T cells OR Beta islet cells OR MEF cells OR Myeloma Cell Lines not otherwise specified OR Other Ovarian Cancer Cell Lines OR Eosinophils OR Other T lymphocytes OR Breast Cancer Cell Lines not otherwise specified OR Sciatic Nerve OR COLO205 OR Other Peripheral blood leukocytes OR Liver OR Hepatocytes OR Smooth muscle cells not otherwise specified OR White Matter OR Pheochromocytoma cell lines not otherwise specified OR Skeletal Muscle OR HCT-15 OR RKO OR U937 OR EKVX OR MOLT-4 OR SNB-75 OR Immature monocyte-derived dendritic cells OR Other Pancreatic Cancer Cell Lines OR Organ Systems not otherwise specified OR Lung Cancer Cell Lines not otherwise specified OR Spinal Cord OR RXF-393 OR BDCA-3+ dendritic cells OR Chondrocytes OR 786-0 OR Choroid Plexus OR Tissues and Primary Cells not otherwise specified OR Hematopoietic progenitor cells OR Salivary Gland OR Astrocytes OR Other Prostate Cancer Cell Lines OR P19 OR RPMI-8266 OR Brainstem OR Intraepithelial T lymphocytes OR Central memory cytotoxic T cells OR Other Monocyte-derived dendritic cells OR Small Intestine OR Bone marrow-derived dendritic cells OR Min6 OR SK-N-SH OR Other Bone marrow cells OR A2780 OR Other Cells OR Neurons not otherwise specified OR BT-474 OR NCI-H23 OR SK-MEL-5) AND

(mol. types = biologic drug OR canonical pathway OR chemical - endogenous mammalian OR chemical - endogenous non-mammalian OR chemical - kinase inhibitor OR chemical - other OR chemical - protease inhibitor OR chemical drug OR chemical reagent OR chemical toxicant OR complex OR cytokine OR disease OR enzyme OR function OR fusion gene/product OR G-protein coupled receptor OR group OR growth factor OR ion channel OR kinase OR ligand-dependent nuclear receptor OR mature microRNA OR microRNA OR other OR peptidase OR

(c) 2000-2024 QIAGEN. All rights reserved.

INGENUITY PATHWAY ANALYSIS
Disease Models & Mechanisms • Supplementary information

Summary of Analysis - A vs D IPA 1-25-24 - 2024-01-25 11:26 AM

phosphatase OR transcription regulator OR translation regulator OR transmembrane receptor OR transporter) AND (data sources = An Open Access Database of Genome-wide Association Results OR BIND OR BioGRID OR Catalogue Of Somatic Mutations In Cancer (COSMIC) OR Chemical Carcinogenesis Research Information System (CCRIS) OR Clinical Genome Resource (ClinGen) OR ClinicalTrials.gov OR ClinVar OR Cognia OR DIP OR DrugBank OR Gene Ontology (GO) OR GVK Biosciences OR Hazardous Substances Data Bank (HSDB) OR HumanCyc OR Ingenuity Expert Findings OR Ingenuity ExpertAssist Findings OR IntAct OR Interactome studies OR MIPS OR miRBase OR miRecords OR Mouse Genome Database (MGD) OR Obesity Gene Map Database OR Online Mendelian Inheritance in Man (OMIM) OR Reactome OR TarBase OR TargetScan Human OR TargetScan Mouse)

Top Canonical Pathways

Name	p-value	Overlap
**Glutamate binding, activation of AMPA receptors and synaptic plasticity**	1.24E-09	15.6 % 5/32
**EPH-Ephrin signaling**	6.21E-09	6.4 % 6/94
**L1CAM interactions**	3.46E-08	4.8 % 6/125
**GABA Receptor Signaling**	4.79E-08	4.5 % 6/132
**Beta-catenin independent WNT signaling**	4.75E-07	4.9 % 5/102

Top Upstream Regulators

Upstream Regulators

Name	p-value	Predicted Activation
**MAPT**	4.49E-11	
**RTN4**	5.10E-10	

(c) 2000-2024 QIAGEN. All rights reserved.

INGENUITY PATHWAY ANALYSIS

Disease Models & Mechanisms • Supplementary information

**memantine**	1.44E-08
**PSEN1**	9.62E-08
**PLX5622**	1.00E-07

Causal Network

Name	p-value	Predicted Activation
**RTN4**	8.93E-09	
**PLX5622**	8.84E-08	
**5-methyltetrahydrofolic acid**	5.43E-07	Inhibited
**MECP2:SIN3A:HDAC1:BDNF gene**	5.64E-07	
**memantine**	5.79E-07	

Top Diseases and Bio Functions

Diseases and Disorders

Name	p-value range	# Molecules
**Neurological Disease**	4.98E-02 - 7.09E-09	34
**Organismal Injury and Abnormalities**	4.99E-02 - 7.09E-09	36
**Developmental Disorder**	4.53E-02 - 8.02E-08	26
**Hereditary Disorder**	4.79E-02 - 8.02E-08	22
**Infectious Diseases**	4.95E-02 - 6.84E-07	17

(c) 2000-2024 QIAGEN. All rights reserved.

Disease Models & Mechanisms • Supplementary information

Summary of Analysis - A vs D IPA 1-25-24 - 2024-01-25 11:26 AM

Molecular and Cellular Functions

Name	p-value range	# Molecules
Cellular Assembly and Organization	4.98E-02 - 2.45E-09	30
Cell Morphology	4.98E-02 - 3.60E-09	26
Cellular Function and Maintenance	4.98E-02 - 7.47E-09	31
Cellular Development	4.93E-02 - 2.51E-08	23
Cellular Growth and Proliferation	4.93E-02 - 2.51E-08	23

Physiological System Development and Function

Name	p-value range	# Molecules
Nervous System Development and Function	4.79E-02 - 3.19E-11	24
Organismal Development	4.93E-02 - 3.19E-11	23
Tissue Morphology	4.79E-02 - 1.06E-10	19
Organ Morphology	4.79E-02 - 1.40E-10	16
Tissue Development	4.79E-02 - 2.51E-08	20

Top Tox Functions

Assays: Clinical Chemistry and Hematology

Name	p-value range	# Molecules
Increased Levels of Creatinine	1.00E-01 - 1.00E-01	1

(c) 2000-2024 QIAGEN. All rights reserved.

INGENUITY PATHWAY ANALYSIS

Disease Models & Mechanisms • Supplementary information

Summary of Analysis - A vs D IPA 1-25-24 - 2024-01-25 11:26 AM

Cardiotoxicity

Name	p-value range	# Molecules
**Cardiac Dilation**	1.20E-01 - 1.53E-03	1
**Cardiac Enlargement**	2.56E-01 - 1.53E-03	2
**Heart Failure**	2.31E-01 - 1.53E-03	3
**Cardiac Dysfunction**	1.20E-01 - 1.36E-02	2
**Cardiac Arrythmia**	9.35E-02 - 1.45E-02	3

Hepatotoxicity

Name	p-value range	# Molecules
**Liver Hyperplasia/Hyperproliferation**	1.70E-01 - 6.54E-05	24
**Liver Inflammation/Hepatitis**	1.44E-01 - 3.61E-04	4
**Hepatocellular carcinoma**	1.70E-01 - 7.63E-03	7
**Liver Steatosis**	2.63E-01 - 7.24E-02	2
**Liver Failure**	1.25E-01 - 1.25E-01	1

Nephrotoxicity

Name	p-value range	# Molecules
**Nephrosis**	6.09E-02 - 4.66E-03	3
**Renal Necrosis/Cell Death**	3.97E-01 - 7.63E-03	4

(c) 2000-2024 QIAGEN. All rights reserved.

INGENUITY PATHWAY ANALYSIS
Disease Models & Mechanisms • Supplementary information

Summary of Analysis - A vs D IPA 1-25-24 - 2024-01-25 11:26 AM

**Glomerular Injury**	1.72E-01 - 9.15E-03	2
**Renal Inflammation**	3.32E-02 - 9.15E-03	1
**Renal Nephritis**	3.32E-02 - 9.15E-03	1

Top Regulator Effect Networks

ID	Regulators	Disease & Functions	Consistency Score
**1**	BHLHE40,DSCAM,KCNJ2	Cell proliferation of tumor cell lines (+3 more)	5.376
**2**	BHLHE40	Cell movement	-4.919

Top Networks

ID	Associated Network Functions	Score
**1**	Nervous System Development and Function, Organ Morphology, Organismal Development	52
**2**	Cardiovascular Disease, Cell-To-Cell Signaling and Interaction, Cellular Compromise	18
**3**	Cellular Function and Maintenance, Cellular Assembly and Organization, Nervous System Development and Function	15

(c) 2000-2024 QIAGEN. All rights reserved.

INGENUITY PATHWAY ANALYSIS
Disease Models & Mechanisms • Supplementary information

Summary of Analysis - A vs D IPA 1-25-24 - 2024-01-25 11:26 AM

**4**	Cellular Function and Maintenance, Molecular Transport, Protein Trafficking	8

Top Tox Lists

Name	p-value	Overlap
**Decreases Permeability Transition of Mitochondria and Mitochondrial Membrane**	1.07E-02	14.3 % 1/7
**Mitochondrial Dysfunction**	1.69E-02	0.8 % 3/355
**Renal Necrosis/Cell Death**	1.96E-02	0.6 % 4/679
**Decreases Depolarization of Mitochondria and Mitochondrial Membrane**	5.37E-02	2.8 % 1/36
**Increases Transmembrane Potential of Mitochondria and Mitochondrial Membrane**	7.38E-02	2.0 % 1/50

Top My Lists

Name	p-value	Overlap
**1.5 fold cutoff A VS D**	2.67E-89	62.3 % 33/53

Top My Pathways

Top ML Disease Pathways

(c) 2000-2024 QIAGEN. All rights reserved.

INGENUITY PATHWAY ANALYSIS
Disease Models & Mechanisms • Supplementary information

Summary of Analysis - A vs D IPA 1-25-24 - 2024-01-25 11:26 AM

Name	p-value	Overlap
**Infection by Dengue virus 2**	5.45E-05	6.2 % 3/48
**Autoimmune glomerulonephritis**	5.17E-04	9.1 % 2/22
**Immune-mediated nephritis**	5.17E-04	9.1 % 2/22
**Axonal neuropathy**	8.41E-04	7.1 % 2/28
**Infection by hepatitis B virus**	1.10E-03	6.2 % 2/32

Top Analysis-Ready Molecules

Expr Fold Change

Expr Fold Change

Molecules	Expr. Value	Chart
**HAP1**	↓ -3.594	
**SRGAP3**	↓ -3.413	
**HECTD4**	↓ -3.062	
**BCR**	↓ -2.810	
**AHI1**	↓ -2.579	
**NDEL1**	↓ -2.526	
**GABBR2**	↓ -2.525	
**DYNC1H1**	↓ -2.518	
**KCNH7**	↓ -2.275	
**MAP1A**	↓ -2.186	

(c) 2000-2024 QIAGEN. All rights reserved.

INGENUITY PATHWAY ANALYSIS

Disease Models & Mechanisms • Supplementary information
